# Extracellular Vesicles from Compression‐Loaded Cementoblasts Promote the Tissue Repair Function of Macrophages

**DOI:** 10.1002/advs.202402529

**Published:** 2024-08-05

**Authors:** Yuhui Yang, Hao Liu, Kunyao Guo, Qianyao Yu, Yi Zhao, Jiayi Wang, Yiping Huang, Weiran Li

**Affiliations:** ^1^ Department of Orthodontics Peking University School and Hospital of Stomatology Beijing 100081 P. R. China; ^2^ National Center for Stomatology & National Clinical Research Center for Oral Diseases & National Engineering Laboratory for Digital and Material Technology of Stomatology & Beijing Key Laboratory for Digital Stomatology & Research Center of Engineering and Technology for Computerized Dentistry Ministry of Health & NMPA Key Laboratory for Dental Materials Beijing 100081 P. R. China

**Keywords:** compression, efferocytosis, extracellular vesicles, polarization, rab35

## Abstract

Treatment strategies for hard tissue defects aim to establish a mineralized microenvironment that facilitates tissue remodeling. As a mineralized tissue, cementum shares a similar structure with bone and exhibits an excellent capacity to resist resorption under compression. Macrophages are crucial for mineralized remodeling; however, their functional alterations in the microenvironment of cementum remain poorly understood. Therefore, this study explores the mechanisms by which cementum resists resorption under compression and the regulatory roles of cementoblasts in macrophage functions. As a result, extracellular vesicles from compression‐loaded cementoblasts (Comp‐EVs) promote macrophage M2 polarization and enhance the clearance of apoptotic cells (efferocytosis) by 2‐ to 3‐fold. Local injection of Comp‐EVs relieves cementum destruction in mouse root resorption model by activating the tissue repair function of macrophages. Moreover, Comp‐EV‐loaded hydrogels achieve significant bone healing in calvarial bone defect. Unexpectedly, under compression, EV secretion in cementoblasts is reduced by half. RNA‐Seq analysis and verification reveal that *Rab35* expression decreases by 60% under compression, thereby hampering the release of EVs. Rab35 overexpression is proposed as a modification of cementoblasts to boost the yield of Comp‐EVs. Collectively, Comp‐EVs activate the repair function of macrophages, which will be a potential therapeutic strategy for hard tissue repair and regeneration.

## Graphical abstract

Inspired by anti‐resorption capability of cementum covering the tooth root, extracellular vesicles derived from compression‐loaded cementoblasts (Comp‐EVs) are isolated and analyzed. Comp‐EVs promote macrophage M2‐type polarization and efferocytosis, while facilitating tissue mineralization extending from cementum to bone. Rab35 is a crucial regulator of Comp‐EV release and serves as an effective target for enhancing Comp‐EV production.

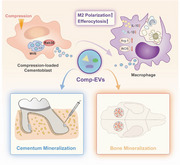

## 1 Introduction

Hard tissue defects are a prominent destructive feature of trauma, tumors, and inflammatory diseases that threaten the health and quality of life of many people worldwide.^[^ ^1^, ^2^ ^]^ Repairing hard tissue has always been challenging, and current treatments have shown limited success; therefore, innovation in therapeutic strategies for hard tissue mineralization and regeneration is critical. Cementum, a mineralized layer covering the tooth root, has a structure and composition similar to bone.^[^ ^3^ ^]^ Cementoblasts, which lie on the surface of the cementum and secrete mineralized matrix, are crucial for the formation of reparative cementum for tissue repair and regeneration.^[^ ^3^, ^4^ ^]^ Interestingly, the resorption of bone and cementum follows a similar process;^[^ ^5^ ^]^ however, when subjected to compression, the cementum is relatively resistant to resorption compared with bone. Osteoclastogenesis of the alveolar bone is rapidly initiated on the compression side of the roots; however, only a small number of osteoclasts adhere to the surface of the cementum. Inspired by the unique anti‐resorption capability of cementum, we aimed to explore the underlying mechanism by which cementum resists resorption under compression, which could guide the development of novel strategies for hard tissue repair.

In the compression‐loaded local microenvironment of the tooth roots, aseptic inflammation occurs in the periodontium, and multiple cell types participate in the regulation of cementum remodeling, including cementoblasts, cementocytes, macrophages, osteoclasts, and periodontal ligament stem cells.^[^ ^6^, ^7^, ^8^, ^9^ ^]^ Macrophages, essential cells in innate immunity, are recruited and activated into various functional types to maintain tissue homeostasis.^[^ ^10^ ^]^ M1‐type macrophages release inflammatory factors that promote inflammatory responses and osteoclastogenesis, while M2‐type macrophages release anti‐inflammatory agents that facilitate tissue repair and regeneration.^[^ ^11^ ^]^ Macrophages are a major member of the phagocyte family; their phagocytic ability is a key function.^[^ ^12^ ^]^ Efferocytosis, the mechanism by which macrophages clear apoptotic cells, is crucial for clearing dead cells and recruiting progenitor cells to resolve inflammation and promote tissue remolding.^[^ ^13^ ^]^ Efferocytosis effectively triggers macrophages to switch to the M2 phenotype by producing anti‐inflammatory factors.^[^ ^14^, ^15^ ^]^ Accumulating evidence supports the notion that intercellular communication between macrophages and tissue‐resident cells is important for microenvironmental remolding.^[^ ^16^, ^17^ ^]^ However, whether and how the interaction between cementoblasts and macrophages contributes to hard tissue mineralization remains unclear.

Extracellular vesicles (EVs) are nano‐sized vesicles encased by bilayer membranes with diameters ranging from 30 to 200 nm.^[^ ^18^, ^19^ ^]^ EVs can be released from various cell types and function as critical intercellular communicators containing biological components such as nucleic acids, proteins, and lipids.^[^ ^20^ ^]^ Growing evidence suggests that macrophages enable the efficient uptake of EVs.^[^ ^21^ ^]^ Moreover, the delivery of EV contents plays a role in the alteration of macrophage functions, such as macrophage polarization, cytokine production, and phagocytic capacity.^[^ ^14^, ^22^ ^]^ Mesenchymal stem cell‐derived EVs exert repair effects via the macrophage‐mediated resolution of inflammation, angiogenesis, and mineralization.^[^ ^23^, ^24^, ^25^ ^]^ Boosting the yield of EVs for easier acquisition and superior biological effectiveness is a promising approach in tissue repair engineering and regenerative medicine.^[^ ^26^, ^27^ ^]^ However, the effects of EVs derived from compression‐loaded cementoblasts on macrophage function remain unclear.

In this study, we evaluated the intercellular effects of compression‐loaded cementoblasts on the tissue repair function of macrophages, including polarization and efferocytosis. Our results revealed that EVs derived from compression‐loaded cementoblasts (Comp‐EVs) are pivotal for macrophage‐mediated hard tissue mineralization. Furthermore, we explored the mechanism of EV secretion from compression‐loaded cementoblasts and developed a strategy to promote the production of Comp‐EVs. Our study provides novel insights into the mechanism underlying anti‐resorption capability of cementum under compression and proposes that Comp‐EVs are a promising strategy for hard tissue repair and regeneration.

## 2 Results

### 2.1 Compression‐Loaded Cementoblasts Promote the M2 Polarization of Macrophages

**Figure 1:**
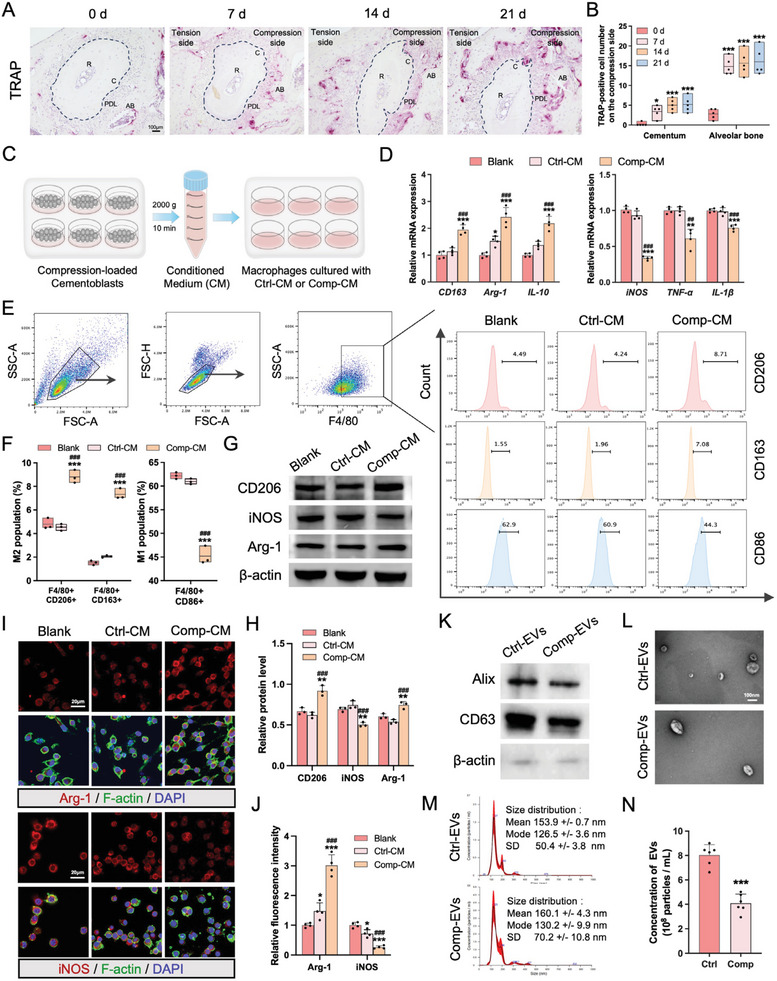
Compression‐loaded cementoblasts promote the M2‐type polarization of macrophages. A) Osteoclasts at the cementum and alveolar bone on the compression side of the tooth roots were stained with Tartrate‐resistant acid phosphatase (TRAP) at days 7, 14, 21. The dashed lines show the outline of the cross section of roots. R, root; C, cementum; PDL, periodontal ligament; AB, alveolar bone. Scale bar = 100 µm. B) Quantification of TRAP‐positive cell number at the compression‐loaded cementum and alveolar bone (n = 5). C) Schematic illustration of conditioned media (CM) collection from cementoblasts and subsequent treatment of macrophages (RAW 264.7). D) Relative mRNA expression levels of macrophage polarization‐related markers (M2: *CD163, Arg‐1, IL‐10*; M1: *iNOS, TNF‐α, IL‐1β*) in macrophages cultured with blank media, Ctrl‐CM, or Comp‐CM for 24 h (n = 4). E) Representative flow cytometric images illustrating the expression of M2 population markers (CD206+ or CD163+) and M1 population marker (CD86+) in F4/80+ macrophages. F) Quantification of flow cytometry analysis showing the percentage of CD206+, CD163+, and CD86+ macrophages (n = 3). G) The protein levels of CD206, Arg‐1 and iNOS in macrophages. 𝛽‐actin served as an internal control for equal loading. H) Semiquantitative analysis in terms of the band intensity (n = 3). I) Representative immunofluorescence images of Arg‐1 and iNOS in macrophages. Nuclei were stained with DAPI, macrophages were labeled with F‐actin. Scale bar = 20 µm. J) Semiquantitative analysis of Arg‐1 and iNOS fluorescent intensity (n = 4). K) Western blotting analysis of EV markers Alix and CD63, and the cytoskeletal protein 𝛽‐actin (n = 3). L) TEM visualization of Ctrl‐EVs and Comp‐EVs morphology (n = 3). Scale bar = 100 nm. M) The size distribution of Ctrl‐EVs and Comp‐EVs as determined by NTA. N) The concentration of Ctrl‐EVs and Comp‐EVs determined by NTA (n = 6). The data are presented as the mean ± SD. *p < 0.05, **p < 0.01, and ***p < 0.001 versus blank group; **^#^** *p* < 0.05, **^##^** *p* < 0.01, and **^###^** *p* < 0.001 versus Ctrl‐CM group.

We compared the resorption activity of compression‐loaded cementum and alveolar bone in a mouse model of tooth movement for different durations. The results of TRAP staining showed that more osteoclasts and resorption lacunae were observed on the surface of alveolar bone, while fewer were present on the cementum surface (**Figure** **1**A,B). Given that osteoclasts are specialized multinucleated cells that differentiate from macrophages,^[^ ^28^ ^]^ we investigated whether compression‐loaded cementoblasts regulate macrophages. A mechanical compression‐loading system was applied, wherein cementoblasts were stimulated with continuous compression (Figure S1A, Supporting Information). We measured the viability and apoptosis of cementoblasts subjected to different compression levels (0, 0.5, 1, 1.5, 2, and 3 g cm^−^^2^) or durations (0, 2, 6, 12, 18, and 24 h). The CCK‐8 assay revealed decreased cell viability, and Annexin V‐FITC/PI staining showed increased apoptosis in cementoblasts subjected to heavy or prolonged compression (Figure S1B–D, Supporting Information). To mimic the physiological force applied in vivo,^[^ ^29^ ^]^ compression at 1.5 g cm^−^^2^ for 12 h was used to cementoblasts in subsequent experiments.

To determine the macrophage polarization state, we treated macrophages (RAW264.7) with conditioned media from compression‐loaded cementoblasts (Comp‐CM) and control cementoblasts (Ctrl‐CM) for 24 h (Figure 1C). The mRNA expressions of representative M2 markers (*CD163*, *Arg‐1*, and *IL‐10*) were increased, while M1 markers (*iNOS*, *TNF‐α*, and *IL‐1β*) were decreased in the group treated with Comp‐CM compared to the Ctrl‐CM and blank groups (Figure 1D). To detect the M1 and M2 populations, macrophages were labeled with F4/80, with the M1 population labeled with CD86, and the M2 population labeled with CD206 or CD163. An increase in the M2 population, and a decrease in the M1 population were observed with the treatment of Comp‐CM (Figure 1E,F). The increased protein levels of CD206 and Arg‐1, along with the decreased iNOS level were confirmed in macrophages in the Comp‐CM group via western blotting (Figure 1G,H). Moreover, immunofluorescence staining showed that Comp‐CM promoted Arg‐1 expression but reduced iNOS expression (Figure 1I,J).

It is worth noting that treatment with Comp‐CM partially inhibited the increased mRNA expression of *iNOS, IL‐1β, and TNF‐α* in LPS and IFN‐γ‐induced macrophages (Figure S2A, Supporting Information). Flow cytometry and western blotting analyses revealed that LPS and IFN‐γ stimulation induced an increase of M1 population and a decrease of M2 population, which were subsequently partially reversed after Comp‐CM treatment (Figure S2B,C, Supporting Information). Treatment with Comp‐CM enhanced the mRNA expressions of M2 markers in macrophages activated by IL‐4 and IL‐13 stimulation (Figure S2D, Supporting Information). The percentage of CD206‐positive cells increased in IL‐4 and IL‐13‐induced macrophages and further increased after Comp‐CM treatment (Figure S2E, Supporting Information).

As major functional effectors of the CM, extracellular vesicles were isolated from both Comp‐CM and Ctrl‐CM through differential ultracentrifugation. Figure S3  (Supporting Information) illustrates the detailed isolation process for extracellular vesicles derived from compression‐loaded cementoblasts (Comp‐EVs) and control cementoblasts (Ctrl‐EVs). Western blotting showed that the Ctrl‐EVs and Comp‐EVs were enriched in the EV markers CD63 (transmembrane) and Alix (cytosolic), while showing negligible expression of the cytoskeletal protein β‐actin (Figure 1K). Transmission electron microscopy (TEM) revealed that the purified vesicles possess a lipid bilayer membrane and exhibit a cup‐shaped morphology (Figure 1L). Nanoparticle tracking analysis (NTA) showed that Ctrl‐EVs and Comp‐EVs displayed similar size distributions, ranging from 50 to 200 nm, consistent with small EVs (Figure 1M). Additionally, NTA revealed that the number of EVs was lower in the Comp‐CM group compared to the Ctrl‐CM group (Figure 1N).

### 2.2 EVs Derived from Compression‐Loaded Cementoblasts Polarize Macrophages into the M2 Phenotype

**Figure 2:**
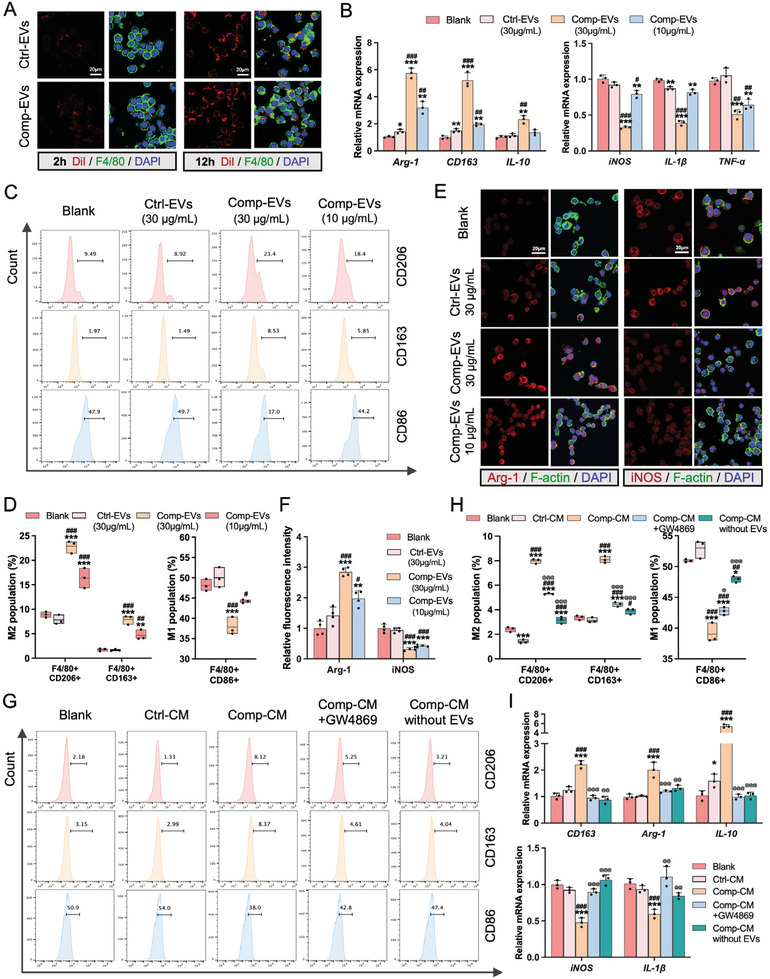
Extracellular vesicles derived from compression‐loaded cementoblasts mediate macrophage M2 polarization. A) Uptake of Ctrl‐EVs and Comp‐EVs by RAW 264.7 macrophages for 2 h or 12 h (n = 3). EVs were labeled with DiI, macrophages were labeled with F4/80. Scale bar = 20 µm. B) qRT‐PCR analysis of M2 and M1 markers in macrophages treated with PBS, Ctrl‐EVs (30 µg mL^−1^), Comp‐EVs (30 µg mL^−1^), or Comp‐EVs (10 µg mL^−1^) for 24 h (n = 3). C) Flow cytometry analysis of M2 and M1 populations in F4/80+ macrophages. D) Quantification of the percentage of CD206+, CD163+, and CD86+ macrophages (n = 3). E) Representative immunofluorescence images of Arg‐1 and iNOS in macrophages. Scale bar = 20 µm. F) Semiquantitative analysis of Arg‐1 and iNOS fluorescent intensity (n = 4). G) Flow cytometry analysis of M2 and M1 populations in macrophages cultured with blank media, Ctrl‐CM, Comp‐CM, Comp‐CM+GW4869, or Comp‐CM without EVs for 24 h. H) Quantification of the percentage of CD206+, CD163+, and CD86+ macrophages (n = 3). I) Relative mRNA expression levels of *CD163, Arg‐1, IL‐10, iNOS*, and *IL‐1β* in macrophages. The data are presented as the mean ± SD. *p < 0.05, **p < 0.01, and ***p < 0.001 versus blank group; **^#^** *p* < 0.05, **^##^** *p* < 0.01, and **^###^** *p* < 0.001 versus Ctrl‐EVs or Ctrl‐CM group; **^@^** *p* < 0.05, **^@@^** *p* < 0.01, and **^@@@^** *p* < 0.001 versus Comp‐CM group.

To confirm the effects of Comp‐EVs on macrophages (RAW264.7), we first traced the uptake of red fluorescence‐labeled EVs, and observed that both Ctrl‐EVs and Comp‐EVs were internalized by macrophages after 2 h, and significantly internalized after 12 h (**Figure** **2**A). Macrophages treated with Ctrl‐EVs and Comp‐EVs (10, 30, 60 µg mL^−1^) showed >95% cell viability, while the cell viability decreased when the concentration of EVs reached 100 µg mL^−1^ (Figure S4A, Supporting Information). The proliferation of macrophages increased in the Ctrl‐EVs and Comp‐EVs group compared to the blank group, with the EV concentration of 30 µg mL^−1^ exhibiting better performance than that of 10 µg mL^−1^ (Figure S4B, Supporting Information). Next, macrophages were treated with Comp‐EVs at relatively high (30 µg mL^−1^) or low (10 µg mL^−1^) concentration for 24 h. qRT‐PCR analysis showed that, compared to the blank and Ctrl‐EVs (30 µg mL^−1^) groups, the expression of M1 markers in the macrophages was reduced, whereas the expression of M2 markers was increased in the high‐concentration Comp‐EVs group; this effect was attenuated in the low‐concentration Comp‐EVs group (Figure 2B). The proportion of M2 macrophages was significantly higher in the Comp‐EVs groups than that in the blank and Ctrl‐EVs groups, with Comp‐EVs (30 µg mL^−1^) group exhibiting the most pronounced elevation (Figure 2C,D). Immunofluorescence staining showed that the expression of Arg‐1 increased, whereas that of iNOS decreased in macrophages treated with Comp‐EVs (Figure 2E,F). Western blotting revealed that the protein levels of CD206 and Arg‐1 increased, while the level of iNOS decreased in the Comp‐EVs group (Figure S5, Supporting Information). Furthermore, treatment with Comp‐EVs partially attenuated M1 polarization in macrophages activated by LPS and IFN‐γ stimulation (Figure S6A,B, Supporting Information), and further promoted M2 polarization in IL‐4 and IL‐13‐induced macrophages (Figure S6C,D, Supporting Information).

Next, we blocked EV secretion using GW4869 or removed EVs from Comp‐CM. Flow cytometry results showed that GW4869 treatment and the removal of EVs from Comp‐CM attenuated M2 polarization in macrophages induced by Comp‐CM (Figure 2G,H). In addition, the mRNA levels of *CD163*, *IL‐10*, and *Arg‐1* were reduced, whereas those of *iNOS* and *IL‐1β* were increased in macrophages treated with Comp‐CM+GW4869 or Comp‐CM without EVs compared to those treated with Comp‐CM (Figure 2I). These results suggest that the presence of EVs in Comp‐CM is crucial for promoting M2 polarization in macrophages.

### 2.3 EVs Derived from Compression‐Loaded Cementoblasts Enhance Efferocytosis in Macrophages

**Figure 3:**
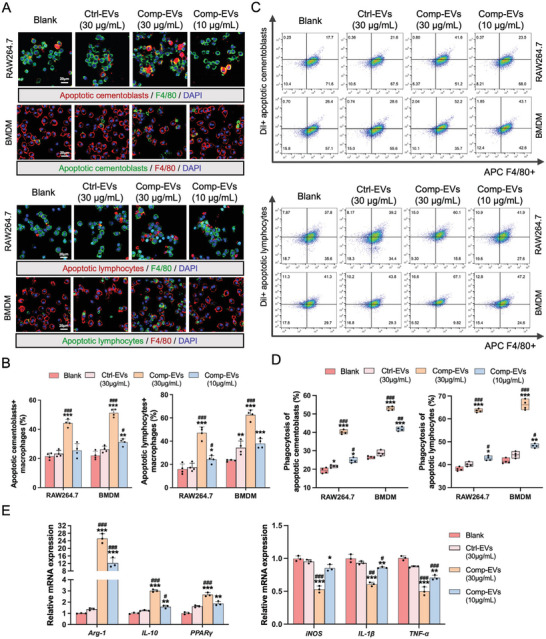
Extracellular vesicles derived from compression‐loaded cementoblasts facilitate efferocytosis and anti‐inflammatory responses in macrophages. A) The RAW264.7 macrophage cell line and bone marrow‐derived macrophages (BMDMs) were treated with PBS, Ctrl‐EVs (30 µg mL^−1^), Comp‐EVs (30 µg mL^−1^), or Comp‐EVs (10 µg mL^−1^) for 12 h, and then incubated with DiI‐ or DiO‐labeled apoptotic cementoblasts and lymphocytes for 45 min. Efferocytosis was analyzed by confocal fluorescence microscopy. Scale bar = 20 µm. B) Quantification of the ratio of apoptotic cementoblasts‐ or lymphocytes‐positive macrophages (n = 4). C) Representative flow cytometry plots of DiI+ apoptotic cementoblasts and lymphocytes engulfed by macrophages. D) Quantification of the percentage of DiI+ F4/80+ macrophages (n = 4). E) Relative mRNA expression levels of *Arg‐1, IL‐10, PPARγ, iNOS, TNF‐α*, and *IL‐1β* in macrophages (RAW264.7) 6 h after phagocytizing apoptotic cementoblasts (n = 3). The data are presented as the mean ± SD. *p < 0.05, **p < 0.01, and ***p < 0.001 versus blank group; **^#^** *p* < 0.05, **^##^** *p* < 0.01, and **^###^** *p* < 0.001 versus Ctrl‐EVs group.

Flow cytometry showed that the number of apoptotic cementoblasts largely increased when the cells were subjected to heavy or prolonged compression (Figure S1C,D, Supporting Information). On the compression side of tooth roots in vivo, local sterile inflammation was induced, accompanied by the infiltration of lymphocytes.^[^ ^30^ ^]^ TUNEL staining showed a significant increase in the number of apoptotic cells on the compression side of the periodontium during tooth movement (Figure S7A, Supporting Information). Since the accumulation of apoptotic cells in compression‐loaded cementum results in the disturbance of tissue remodeling, these findings prompted us to evaluate whether Comp‐EVs regulate macrophage efferocytosis. Cementoblasts and T lymphocytes were treated with staurosporine (STS) to induce apoptosis (Figure S7B, Supporting Information). We conducted the efferocytosis assays using both the mouse macrophage RAW264.7 cell line and mouse bone marrow‐derived macrophages (BMDMs). Macrophages were incubated with DiI‐ or DiO‐labeled apoptotic cementoblasts and lymphocytes for 45 min. Comp‐EVs increased the overall efferocytosis capacity of macrophages, as the number of apoptotic cementoblasts‐ or lymphocyte‐containing macrophages increased in the Comp‐EVs group compared to the Ctrl‐EVs and blank groups, especially at relatively high concentrations (**Figure** **3**A,B). BMDMs continuously phagocytosed more apoptotic cells than RAW264.7 macrophages after the treatment of Comp‐EVs. We then screened macrophages from unengulfed apoptotic cells after gating F4/80+ and DiI+ cells (Figure S8, Supporting Information). Flow cytometry results showed that DiI+ F4/80+ macrophages were significantly enriched in the Comp‐EVs group (Figure 3C,D). Furthermore, the mRNA levels of anti‐inflammatory factors *Arg‐1*, *IL‐10*, and *PPARγ* were increased in the Comp‐EVs group exposed to apoptotic cementoblasts. In contrast, the expression levels of pro‐inflammatory factors *iNOS*, I*L‐1β*, and *TNF‐α* were decreased compared to the Ctrl‐EVs and blank groups (Figure 3E). These results suggest that Comp‐EVs facilitate macrophage efferocytosis, promoting tissue repair.

### 2.4 EVs Derived from Compression‐Loaded Cementoblasts Promote Cementum Repair

**Figure 4:**
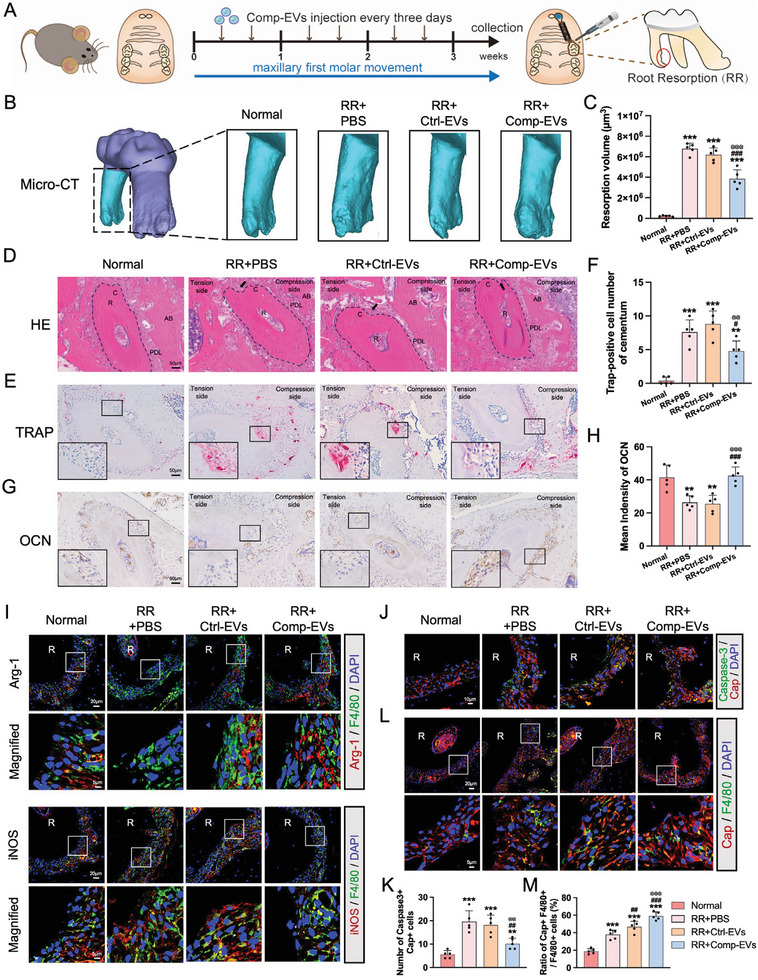
Extracellular vesicles from compression‐loaded cementoblasts promote the repair function of macrophages and alleviate root resorption under compression loading. A) Overview of the experimental design for mouse root resorption. B) Representative 3D Micro‐CT reconstruction images of the maxillary first molar in the normal, RR+PBS, RR+Ctrl‐EVs, and RR+Comp‐EVs groups after tooth movement for 3 weeks. RR, root resorption. C) Quantification of the resorption volume of distobuccal roots (n = 5). D) Representative HE staining images of the distobuccal roots. The black arrow indicates the resorption lacuna. R, root; C, cementum; PDL, periodontal ligament; AB, alveolar bone. Scale bar = 50 µm. E) TRAP staining of osteoclasts on the compression side of distobuccal roots. Scale bar = 50 µm. F) Quantification of TRAP‐positive cell number at the compression‐loaded cementum (n = 5). G) Representative immunohistochemical staining images of OCN. Scale bar = 50 µm. H) Quantitative analysis of OCN‐positive intensity at the compression‐loaded cementum (n = 5). I) Representative immunofluorescence images of Arg‐1 and iNOS staining on the compression side of roots. Macrophages were labeled with F4/80. Scale bar, 20 µm. (n = 5). J,K) Representative immunofluorescence images of apoptotic marker Caspase‐3 and cementum attachment protein (Cap). Scale bar, 10 µm. Counts of Caspase‐3+ Cap+ cells on the surface of the compression‐loaded cementum (n = 5). L) Representative immunofluorescence images show colocalization of F4/80+ macrophages with Cap+ cells. Scale bar, 20 µm. M) Macrophages that stain positive for Cap are scored as undergoing efferocytosis. Quantification of the ratio of Cap+ F4/80+ macrophages to total F4/80+ macrophages (n = 5). The data are presented as the mean ± SD. *p < 0.05, **p < 0.01, and ***p < 0.001 versus normal group; **^#^** *p* < 0.05, **^##^** *p* < 0.01, and **^###^** *p* < 0.001 versus RR+PBS group; **^@^** *p* < 0.05, **^@@^** *p* < 0.01, and **^@@@^** *p* < 0.001 versus RR+Ctrl‐EVs group.

To verify the tissue repair capacity of Comp‐EVs on the cementum, a prolonged tooth movement mouse model was established to induce cementum resorption. PBS, Ctrl‐EVs (1.5 µg µL^−1^), and Comp‐EVs (1.5 µg µL^−1^) were locally injected into the buccal side of the compression‐loaded molars every three days (**Figure** **4**A). Based on the results of the micro‐CT analysis, 3D reconstructed images of the roots showed that the resorption volume in the Comp‐EVs group was significantly lower than that in the Ctrl‐EVs and PBS groups (Figure 4B,C). Consistent with the micro‐CT results, hematoxylin‐eosin (HE) staining revealed that the resorption lacunae on the compression side of roots were smaller after injection with Comp‐EVs (Figure 4D). The number of TRAP‐positive osteoclasts on the surface of the compression‐loaded cementum in the Comp‐EVs group was also lower than that in the Ctrl‐EVs and PBS groups (Figure 4E,F). In parallel, the expression of the mineralization‐associated marker osteocalcin (OCN) was increased on the compression side receiving Comp‐EVs (Figure 4G,H).

Double immunofluorescence staining was performed to evaluate the effects of Comp‐EVs on macrophage activation. The results show that the proportion of Arg‐1+ F4/80+ M2‐type macrophages increased, while that of iNOS+ F4/80+ M1‐type macrophages decreased in the Comp‐EVs group compared to the Ctrl‐EVs and PBS groups (Figure 4I). Immunohistochemistry also showed that the Comp‐EVs group expressed higher levels of CD163 and lower levels of iNOS on the compression side of roots (Figure S9, Supporting Information). Furthermore, increased apoptosis of cementum attachment protein (Cap)‐positive cells was observed on the compression side of roots, whereas the number of apoptotic cells decreased with Comp‐EVs injection (Figure 4J,K). The Comp‐EVs group also exhibited a significant increase of Cap+ F4/80+ macrophages, suggesting that Comp‐EVs improved macrophage phagocytosis of apoptotic cells (Figure 4L,M). These results are consistent with the in vitro experiments, indicating the potential of Comp‐EVs in cementum repair by promoting tissue repair function of macrophages.

### 2.5 EVs Derived from Compression‐Loaded Cementoblasts Promote Bone Regeneration

**Figure 5:**
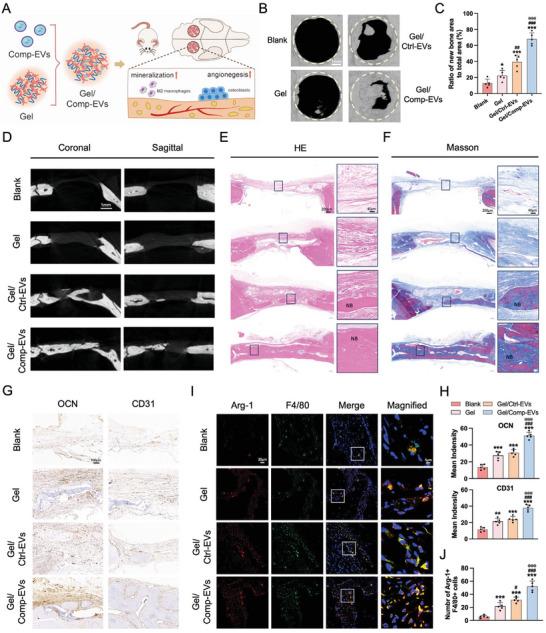
Extracellular vesicles from compression‐loaded cementoblasts promote bone regeneration. A) Schematic diagram of the repair process of rat calvarial bone defects with Gel/Comp‐EVs implantation. B) Representative micro‐CT images of calvarial bone defects in blank, Gel, Gel/Ctrl‐EVs, and Gel/Comp‐EVs groups after 12‐week implantation. The yellow circle indicates the defect boundary. Scale bar = 1 mm. C) Quantification of the ratio of new bone area to total defect area (n = 5). D) Representative micro‐CT images of calvarial bone defects in coronary and sagittal sections. Scale bar = 1 mm. E,F) Representative HE and Masson's trichrome staining images of the defect area. NB, new bone. Scale bar = 200 µm. G) Representative immunohistochemical staining images of OCN and CD31 in the defect area. Scale bar = 100 µm. H) Quantitative analysis of OCN and CD31‐positive intensity (n = 5). I) Representative immunofluorescence images of Arg‐1 and F4/80 staining in newly formed bone area. Scale bar, 20 µm. J) Counts of Arg‐1+ F4/80+ macrophages (n = 5). The data are presented as the mean ± SD. *p < 0.05, **p < 0.01, and ***p < 0.001 versus blank group; **^#^** *p* < 0.05, **^##^** *p* < 0.01, and **^###^** *p* < 0.001 versus Gel group; **^@^** *p* < 0.05, **^@@^** *p* < 0.01, and **^@@@^** *p* < 0.001 versus Gel/Ctrl‐EVs group.

Given the potential of Comp‐EVs for tissue mineralization, a rat calvarial bone defect model was established to explore the repair capacity of Comp‐EVs on bone tissue. Growing evidence has shown that EV‐loaded hydrogels have great potential for tissue repair and bone formation, as they help enhance the delivery and stability of EVs.^[^ ^31^ ^]^ We prepared GelMA hydrogels loaded with Comp‐EVs and Ctrl‐EVs, and investigated their EV release profiles. The results showed that Comp‐EVs and Ctrl‐EVs were continuously released from the hydrogels (Figure S10, Supporting Information). GelMA hydrogels with PBS were used as a control group, and the defect without any implant served as the blank group (**Figure** **5**A). Twelve weeks after implantation, micro‐CT analysis showed that the defect areas were filled with new bone in the Gel/Comp‐EVs group (67.9%), which was much higher than in the Gel/Ctrl‐EVs (40.5%), Gel (23.2%), and blank (13.5%) groups (Figure 5B,C). The BV/TV and BMD values indicated more new bone tissue generation in the Gel/Comp‐EVs group (Figure S11, Supporting Information). Observation of the coronary and sagittal sections further demonstrated that little new bone was formed in the Gel and blank groups. The mineralization effect was limited in the Gel/Ctrl‐EVs group, whereas the Gel/Comp‐EVs group exhibited substantial new bone mineralization (Figure 5D). HE and Masson staining showed the formation of mineralized tissue in the Gel/Comp‐EVs group. Large amounts of residual hydrogels were observed in the Gel/Ctrl‐EVs and Gel groups, while increased mineralized structures with few residual hydrogels were observed in the Gel/Comp‐EVs group (Figure 5E,F). The expression levels of the mineralization‐associated marker OCN and the vascularization‐associated marker CD31 were increased in the Gel/Comp‐EVs group (Figure 5G,H). Furthermore, a higher percentage of Arg‐1+ F4/80+ macrophages was observed in the regenerated tissue transplanted with Gel/Comp‐EVs than in the Gel/Ctrl‐EVs, Gel, and blank groups (Figure 5I,J). These results indicate that, besides promoting cementum, Comp‐EVs also effectively promote bone mineralization and regeneration.

### 2.6 Compression Regulates EV Release in a Rab35‐Dependent Mechanism

**Figure 6:**
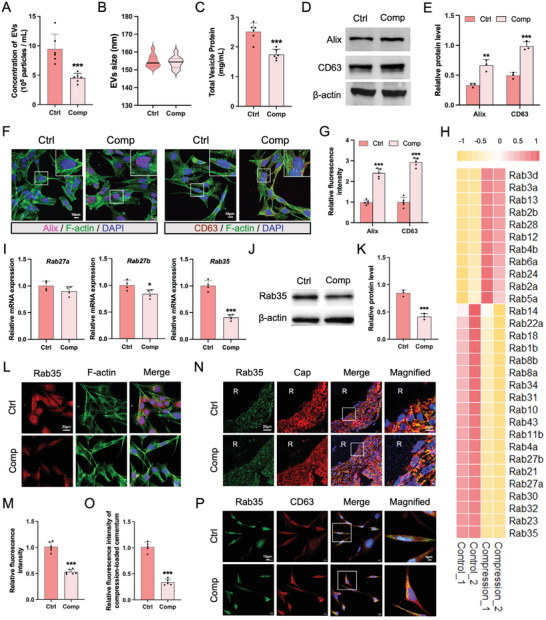
Rab35 is differentially downregulated in compression‐loaded cementoblasts, inhibiting the release of extracellular vesicles. A) The concentration of Ctrl‐EVs and Comp‐EVs released from an equal number of cementoblasts determined by NTA (n = 6). B) Violin plot showing the diameter of Ctrl‐EVs and Comp‐EVs measured by TEM (n = 10). C) The protein concentration of Ctrl‐EVs and Comp‐EVs determined by BCA assay (n = 6). D,E) The protein levels of Alix and CD63 in control and compression‐loaded cementoblasts. Semiquantitative analysis of the band intensity (n = 3). F) Representative immunofluorescence images of Alix and CD63, with F‐actin for cytoskeleton and DAPI for nuclei. Scale bar = 10 µm. G) Semiquantitative analysis of Alix and CD63 fluorescent intensity (n = 5). H) Heatmap diagram of differential RNA expression of Rab GTPase family in control and compression‐loaded cementoblasts (n = 2). I) Relative mRNA expression levels of *Rab27a, Rab27b, Rab35* (n = 4). J,K) The protein level of Rab35 and semiquantitative analysis (n = 3). L,M) Representative immunofluorescence images of Rab35 and quantification of Rab35 fluorescent intensity (n = 6). Scale bar = 20 µm. N,O) Representative immunofluorescence images of Rab35 and Cap on the surface of compression‐loaded cementum and semiquantitative analysis (n = 5). Scale bar = 20 µm. P) Representative immunofluorescence images showing colocalization of CD63 and Rab35 staining in compression‐loaded cementoblasts (n = 4). Scale bar = 10 µm. The data are presented as the mean ± SD. *p < 0.05, **p < 0.01, and ***p < 0.001 versus control group.

While isolating EVs from compression‐loaded cementoblasts, we discovered a large decrease in the number of EVs from Comp‐CM compared with those from Ctrl‐CM (**Figure** **6**A). TEM and NTA analyses showed that Comp‐EVs had a size distribution and morphology similar to those of Ctrl‐EVs (Figure 6B; Figure 1M). The total protein levels of Comp‐EVs also decreased, as confirmed using the BCA assay (Figure 6C). The number and density of collagen fibers deposited in the extracellular matrix (ECM) were not increased in the compression group compared to the control group (Figure S12, Supporting Information), indicating that the release of Comp‐EVs from ECM into the supernatant was not obstructed. Next, we determined whether vesicles accumulated in compression‐loaded cementoblasts by analyzing the expression of vesicle‐related markers. Western blot analysis revealed that the protein levels of CD63 and Alix were concomitantly increased in compression‐loaded cementoblasts (Figure 6D,E). An increased number of Alix‐ and CD63‐positive vesicles were also observed in compression‐loaded cementoblasts (Figure 6F,G), suggesting that compression reduced the release of EVs from cementoblasts.

To promote Comp‐EV secretion, we investigated the mechanism of EV release from compression‐loaded cementoblasts. EVs are generated by the inward budding of the endosomal membranes of multivesicular bodies (MVBs), resulting in the formation of intraluminal vesicles (ILVs), followed by MVB fusion with the plasma membrane to release these vesicles.^[^ ^32^ ^]^ It has been demonstrated that Rab GTPase proteins are required for the motility of MVBs, and facilitate the docking and fusion of MVBs with the cell membrane.^[^ ^33^, ^34^ ^]^ Thus, we screened the members of the Rab GTPase family using our previously published RNA‐Seq data of cementoblasts under compression.^[^ ^35^ ^]^ The results showed that *Rab35* was the most downregulated Rab GTPase gene in response to compression (Figure 6H). qRT‐PCR analysis showed that *Rab35* expression largely decreased in compression‐loaded cementoblasts, whereas the expressions of *Rab27a* and *Rab27b* were only slightly decreased (Figure 6I). Consistent with the gene expression results, compression significantly inhibited the protein level of Rab35 in cementoblasts (Figure 6J–M). Rab35 expression also decreased in Cap+ cells in compression‐loaded cementum (Figure 6N,O). To investigate the subcellular localization of Rab35 in compression‐loaded cementoblasts, we performed a co‐localization analysis of Rab35 and CD63. An increase in the co‐localization of Rab35 with CD63 was observed in compression‐loaded cementoblasts, whereas the overall expression of Rab35 decreased (Figure 6P). These results indicate that compression inhibited the transport of MVBs by regulating the expression and localization of Rab35 in cementoblasts, resulting in decreased EV release.

### 2.7 Rab35 Overexpression Stimulates EV Release in Compression‐Loaded Cementoblasts

**Figure 7:**
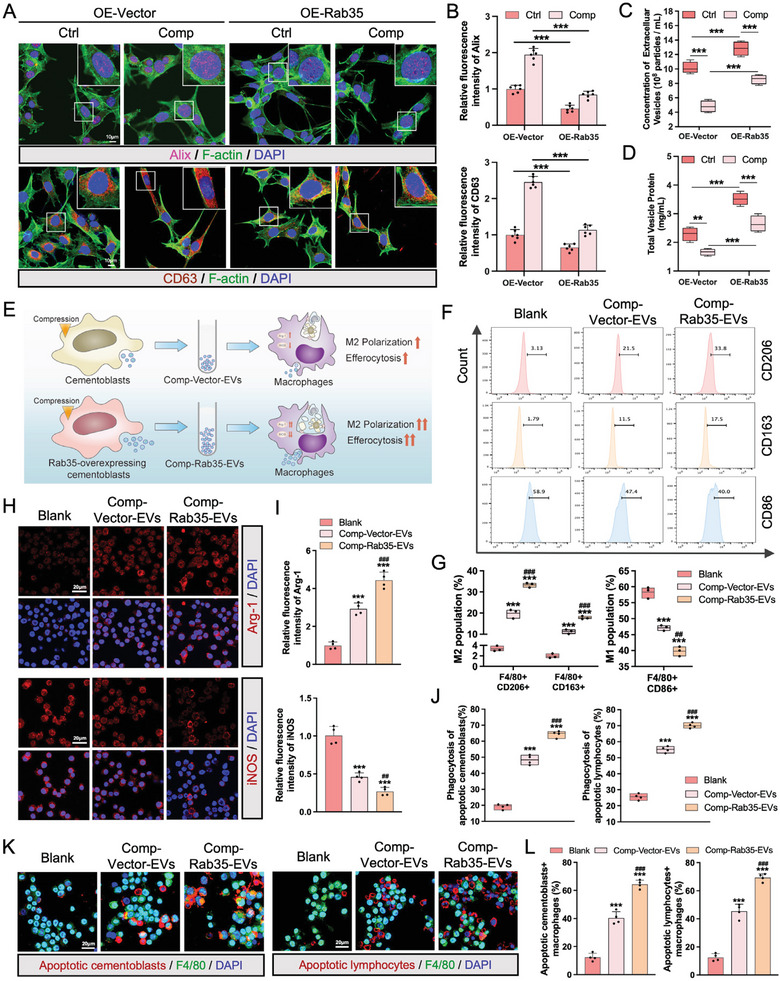
Rab35 overexpression stimulates the release of extracellular vesicles in compression‐loaded cementoblasts. A) Representative immunofluorescence images of Alix and CD63 in control and Rab35‐overexpressing cementoblasts subjected to compression. Scale bar = 10 µm. B) Semiquantitative analysis of Alix and CD63 fluorescent intensity (n = 6). C) The concentration of Ctrl‐EVs and Comp‐EVs isolated from control and Rab35‐overexpressing cementoblasts assessed by NTA (n = 4). D) The protein concentration of Ctrl‐EVs and Comp‐EVs determined by BCA assay (n = 4). E) Schematic of macrophage function alteration activated by Comp‐EVs derived from Rab35‐overexpressing cementoblasts (Comp‐Rab35‐EVs) and vector‐transfected control cementoblasts (Comp‐Vector‐EVs). F) Flow cytometry analysis of M2 and M1 populations in F4/80+ macrophages (RAW264.7) treated with PBS, Comp‐Vector‐EVs, or Comp‐Rab35‐EVs for 24 h. G) Quantification of the percentage of CD206+, CD163+, and CD86+ macrophages (n = 3). H,I) Representative immunofluorescence images of Arg‐1 and iNOS in macrophages and semiquantitative analysis (n = 4). Scale bar = 20 µm. J) Macrophages (RAW264.7) were treated with PBS, Comp‐Vector‐EVs, or Comp‐Rab35‐EVs for 12 h, and then incubated with DiI‐labeled apoptotic cementoblasts or lymphocytes for 45 min. Flow cytometry analysis of the percentages of DiI+ F4/80+ macrophages (n = 4). K) Representative immunofluorescence images of DiI‐labeled apoptotic cementoblasts‐ and lymphocytes engulfed by macrophages. Scale bar = 20 µm. L) Quantification of the ratio of apoptotic cementoblasts‐ or lymphocytes‐positive macrophages (n = 4). The data are presented as the mean ± SD. *p < 0.05, **p < 0.01, and ***p < 0.001 versus blank group; **^#^** *p* < 0.05, **^##^** *p* < 0.01, and **^###^** *p* < 0.001 versus Comp‐Vector‐EVs group.

Because Rab35 is crucial for the release of EVs under compression, we constructed Rab35‐overexpressing cementoblasts to boost the yield of Comp‐EVs. The efficiency of Rab35 overexpressing was confirmed by qRT‐PCR and western blotting analysis (Figure S13A,B, Supporting Information). We then investigated whether Rab35 overexpression in cementoblasts affected cell viability and mineralization capability. The results of CCK‐8 assay showed that the proliferation of Rab35‐overexpressing cementoblasts was similar to that of the control group (Figure S14A, Supporting Information). In addition, the expression of mineralization‐related genes and proteins, as well as ALP activity, was slightly increased in the Rab35‐overexpressing cementoblasts, but these changes were not statistically significant (Figure S14B–E, Supporting Information). Next, we analyzed the effects of Rab35 overexpression on Comp‐EV release. Immunofluorescence staining showed Rab35 overexpression significantly downregulated the accumulation of Alix‐ and CD63‐positive vesicles in compression‐loaded cementoblasts compared to the control group (**Figure** **7**A,B). Concomitantly, Rab35 overexpression stimulated EV release from compression‐loaded cementoblasts (Figure 7C). The protein levels of Comp‐EVs were increased in Rab35‐overexpressing group compared to the control group (Figure 7D).

Given our findings that Comp‐EVs enhanced the repair function of macrophages and that Rab35 promoted Comp‐EV release, we isolated Comp‐EVs from equal numbers of Rab35‐overexpressing cementoblasts (Comp‐Rab35‐EVs) and control cementoblasts (Comp‐Vector‐EVs). As anticipated, the concentration of Comp‐Rab35‐EVs (46 µg mL^−1^) was higher than that of Comp‐Vector‐EVs (30 µg mL^−1^). Next, Comp‐Rab35‐EVs and Comp‐Vector‐EVs, released from an equal number of cementoblasts, were used to treat macrophages to verify the capacity of Comp‐Rab35‐EVs for macrophage activation (Figure 7E). Flow cytometry showed that the percentage of the M2 population in the Comp‐Rab35‐EVs group was higher than that in the Comp‐Vector‐EVs group, and much higher than that in the blank group (Figure 7F,G). Meanwhile, a higher expression of Arg‐1 and a lower expression of iNOS were observed in macrophages treated with Comp‐Rab35‐EVs than in the Comp‐Vector‐EVs and blank groups (Figure 7H,I).

**Figure 8:**
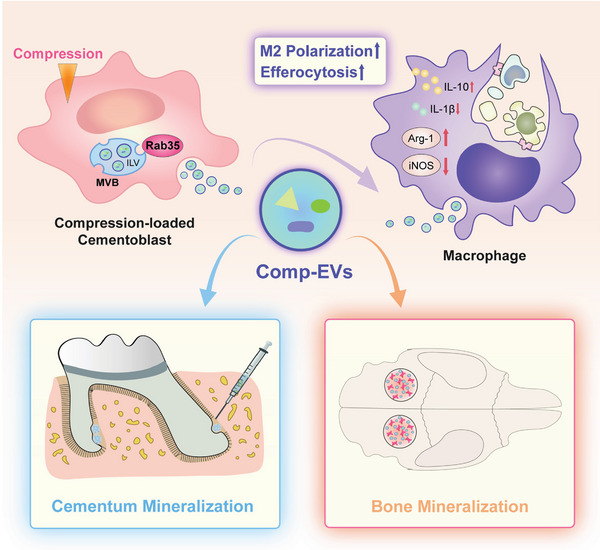
Schematic of extracellular vesicles derived from compression‐loaded cementoblasts (Comp‐EVs) promoting hard tissue mineralization and regeneration by mediating the tissue repair function of macrophages.

Subsequently, we evaluated the effect of Comp‐Rab35‐EVs on the phagocytic capacity of macrophages. Flow cytometry showed that the percentage of the DiI+ F4/80+ macrophage subpopulation was higher in the Comp‐Rab35‐EVs group compared to the Comp‐Vector‐EVs and blank groups (Figure 7J). An increased number of apoptotic cementoblasts or lymphocyte‐containing macrophages was also observed in the Comp‐Rab35‐EVs group compared to the Comp‐Vector‐EVs and blank groups (Figure 7K,L). Taken together, these data establish that Rab35 overexpression efficiently promote EV release in compression‐loaded cementoblasts, which is beneficial for increasing Comp‐EV yield and enhancing the repair function of macrophages (**Figure** **8**).

## 3 Discussion

Remodeling of the periodontium in response to force stimulus is a prerequisite for tooth movement.^[^ ^29^, ^30^ ^]^ Osteoclast activity is induced by mechanical signals on the compression side of roots, whereas the root tissue is relatively resistant to resorption. Therefore, we focused on the intercellular communication between cementoblasts and macrophages to explore the role of EVs from compression‐loaded cementoblasts in hard tissue mineralization. Our study sheds light on the effects of Comp‐EVs on the activation of macrophage repair functions, which are beneficial for promoting the mineralization and regeneration of hard tissue. Our findings revealed that compression inhibited EV release via a Rab35‐dependent mechanism. We further proposed that Rab35 overexpression is a potential strategy to enhance Comp‐EV release and increase Comp‐EV yield for therapeutic applications.

Sterile inflammatory responses have been shown to occur in the compression‐loaded area of the periodontium. The interaction between cementoblasts and immune cells is vital for shaping the cementum tissue mineralization microenvironment. Previous studies have demonstrated the effects of M1‐ or M2‐type macrophages on cementoblast mineralization;^[^ ^8^, ^36^ ^]^ nevertheless, how compression‐loaded cementoblasts regulate macrophage functions remains unexplored. Our study provides evidence that compression‐loaded cementoblasts have a promotive effect on macrophage M2 polarization and highlights that Comp‐EVs exhibited a stronger ability to polarize macrophages into the M2 type compared to Ctrl‐EVs. Notably, EVs isolated from Comp‐CM played a dominant role in macrophage activation, as confirmed by the diminished effect of M2 polarization when macrophages were treated with EV inhibitors and CM without EVs. Previous studies have reported that mechanical force‐stimulated periodontal ligament cells induce osteoclast differentiation and promote macrophage M1 polarization.^[^ ^37^, ^38^ ^]^ In contrast, compression‐loaded cementoblasts exert opposite effects via Comp‐EVs to maintain an anti‐inflammatory microenvironment for cementum repair and stability.

Macrophages are widespread immune cells that phagocytose apoptotic or necrotic cells to dampen inflammatory responses and restore tissue homeostasis.^[^ ^13^, ^39^ ^]^ In this study, we found an increased number of apoptotic cells on the surface of compression‐loaded roots, which may cause an imbalance in tissue mineralization; thus, it is important to identify the regulation of Comp‐EVs in macrophage efferocytosis. Emerging evidence has shown that the dysfunction of macrophage efferocytosis is related to the aggravation of chronic inflammation in the periodontal tissue.^[^ ^40^, ^41^ ^]^ Plasma EVs interact with macrophages and control the inflammatory processes via augmented efferocytosis.^[^ ^14^ ^]^ Our data revealed that Comp‐EVs enhance the phagocytic ability of macrophages. Furthermore, our mouse root resorption model suggested that the injection of Comp‐EVs into the compression side of roots ameliorated root resorption by promoting macrophage repair functions to establish a microenvironment conducive to tissue remodeling and repair. Based on these findings, Comp‐EVs promote macrophage repair functions, offering a promising therapeutic option for hard tissue repair and engineering. We also used a rat calvarial defect model to evaluate the tissue repair capacity of Comp‐EVs in bones. Our results showed that applying GelMA/Comp‐EVs markedly promoted osteogenesis and angiogenesis in the defect area, resulting in favorable bone defect healing. The increased infiltration of M2 macrophages in the defect area indicates that the repair function of Comp‐EVs in bones may be related to the activation of M2 macrophages. Taken together, we uncovered the remarkable capacity of Comp‐EVs to activate macrophage repair functions and promote hard tissue mineralization and regeneration.

Furthermore, we found that EV release from compression‐loaded cementoblasts was inhibited. The promotion of EV release is crucial for unlocking the potential of EV‐based therapy;^[^ ^34^, ^42^ ^]^ thus, it is necessary to investigate the mechanism of EV release in compression‐loaded cementoblasts. EV release is a multistep process involving the transport of MVBs and their docking and fusion with the plasma membrane.^[^ ^43^, ^44^ ^]^ The accumulation of CD63‐positive vesicles in compression‐loaded cementoblasts suggests that compression may act in the later stages of EV biogenesis, such as during the docking or fusion stages of MVBs. Small Rab GTPases have been reported to function in MVBs transport, docking, and fusion and play a regulatory role in EV release.^[^ ^45^, ^46^ ^]^ We identified differentially expressed Rab proteins in compression‐loaded cementoblasts and found that Rab35 expression was significantly downregulated. We also observed the co‐localization of Rab35 and CD63 under compression, suggesting that Rab35 plays a critical role in regulating Comp‐EV release. Growing evidence has shown that Rab35 mediates endocytic membrane trafficking and regulates the docking of endocytic vesicles with the plasma membrane, contributing to MVB fusion with the plasma membrane to secrete EVs.^[^ ^45^, ^47^, ^48^ ^]^ Recently, Rab35 has been reported to target damaged autophagosomes, facilitating autophagosome maturation.^[^ ^49^ ^]^ The activation of mTORC1, which inhibits the autophagic flux, reduces EV release, while the autophagy activator rapamycin stimulates EV release.^[^ ^50^, ^51^, ^52^ ^]^ Interestingly, in our previous published studies, we discovered that autophagy was inhibited in compression‐loaded cementoblasts.^[^ ^7^, ^53^ ^]^ This suggests that the specific mechanisms underlying the interaction between Rab35 and autophagy may be involved in EV release in compression‐loaded cementoblasts.

The overexpression of Rab35 rescued the decreased EV release in compression‐loaded cementoblasts, suggesting it as a potential method to improve the production of Comp‐EVs. Previous studies have reported that chemical and biological remodeling holds great potential for promoting EV secretion.^[^ ^26^ ^]^ Our findings elucidate the mechanisms of Rab35‐dependent EV release in compression‐loaded cementoblasts and provide new insights for the development of related therapeutic strategies. Combining the above results, we compared the effects of Comp‐Rab35‐EVs with those of Comp‐Vector‐EVs released from equal amounts of cementoblasts on the repair function of macrophages. We found that Comp‐Rab35‐EVs exhibited a stronger capacity to promote macrophage repair than Comp‐Vector‐EVs.

Although our results revealed novel discoveries, there are still some limitations. First, compression loading on cementoblasts in vitro does not precisely simulate the local microenvironment in vivo. Second, further investigation is needed to elucidate the contents of Comp‐EVs and identify the cargos responsible for the enhancement of the repair function of macrophages. Third, the mechanism underlying the Comp‐EV‐mediated modulation of macrophage functions remains unexplored and needs to be further determined in future research. Moreover, the development of bioactive materials to achieve the sustained release of EVs for long‐term therapeutic applications is of great value.

## 4 Conclusion

In conclusion, our findings show that EVs from compression‐loaded cementoblasts (Comp‐EVs) promote the repair function of macrophages, including M2‐type polarization and phagocytic capacity for apoptotic cells. The repair ability of Comp‐EVs can also be extended from the cementum to the bone, and various macrophage‐mediated tissue remodeling and regeneration. Furthermore, our data demonstrate that compression inhibits EV release in a Rab35‐dependent manner and propose Rab35 overexpression as a modification to increase the yield of Comp‐EVs for engineering applications. Our study uncovered the repair effects of Comp‐EVs for the treatment of hard tissue defects, promoting the development of immunomodulatory therapies for tissue engineering and regenerative medicine.

## 5 Experimental Section

### Cell Culture

The immortalized murine cementoblast cell line OCCM‐30 was provided by Dr. Martha J. Somerman (National Institutes of Health). To induce cementoblast mineralization, we prepared mineralized medium (MM) consisting of Dulbecco's modified Eagle's medium (DMEM) (Gibco, Thermo Fisher Scientific, Waltham, MA, USA), 10% fetal bovine serum (FBS, Gibco), 1% penicillin‐streptomycin (Gibco), 10 mM sodium β‐glycerophosphate (Sigma‐Aldrich, St. Louis, MO, USA), and 50 µg mL^−1^ ascorbic acid (Sigma‐Aldrich). The murine macrophage cell line RAW 264.7 was purchased from Servicebio Biotechnology Co., Ltd. (Wuhan, Hubei, China). The cells were cultured in DMEM containing 10% FBS and 1% penicillin/streptomycin. Jurkat (human T lymphocyte) cells were purchased from Servicebio Biotechnology Co., Ltd. and cultured in RPMI‐1640 medium (Gibco) supplemented with 10% FBS and 1% penicillin/streptomycin. All cells were cultured in a 5% CO_2_ incubator at 37 °C.

### Primary Cell Culture

Bone marrow‐derived macrophages (BMDMs) were isolated from the femurs and tibias of 8–12‐week‐old mice and cultured in DMEM supplemented with 10% FBS, 1% penicillin/streptomycin, and 50 ng mL^−1^ M‐CSF (Peprotech, USA) for 7 days.

### Macrophage Polarization

The M0 macrophages were polarized into M1 using 200 ng mL^−1^ LPS (Sigma–Aldrich) and 50 ng mL^−1^ IFN‐γ (PeproTech), and polarized into M2 using 200 ng mL^−1^ IL‐4 (PeproTech) and 50 ng mL^−1^ IL‐13 (PeproTech) for 24 h. The induced M1 or M2 macrophages were then treated with conditioned media or EVs for an additional 24 h.

### Application of Compression In Vitro

A mechanical force‐loading device to apply compression in vitro was used. Before static compression, the culture medium was replaced with FBS‐free DMEM. A customized glass cover (32 mm in diameter, 0.8 mm in thickness) was placed over a confluent cell layer in the six‐well plate, and a plastic bottle containing steel balls was placed over the glass cover to apply compression. The magnitude of compression was set at 1.5 g cm^−^^2^ for 12 h based on our previously established protocols.^[^ ^53^ ^]^ Cells cultured without compression served as the control group.

### Cell Apoptosis Assay with Flow Cytometry

Cell apoptosis was detected using the Annexin V‐FITC/PI Apoptosis Detection Kit (Dojindo, Kumamoto, Japan). Briefly, harvested cells were washed twice with PBS and resuspended in 100 µL binding buffer. Subsequently, the cells were stained with 5 µL Annexin V‐FITC and 5 µL PI for 15 min at room temperature in the dark, and then analyzed using flow cytometry (Beckman CytoFLEX FCM, USA).

### Conditioned Media Preparation

Conditioned media (CM) from OCCM‐30 cells were collected following the application of compression and centrifuged at 2000 g for 10 min to remove cellular debris. Ctrl‐CM and Comp‐CM were harvested after filtration through a 0.45‐µm filter (Biosharp, Hefei, China) and used for further experiments.

### RNA Extraction and Quantitative Real‐Time Polymerase Chain Reaction (qRT‐PCR)

RNA extraction was performed using TRIzol reagent (Invitrogen). The quality and concentration of the extracted RNA were determined by measuring the absorbance at 260 and 280 nm using a NanoDrop spectrophotometer (Thermo Fisher Scientific). The mRNA was reverse‐transcribed using a cDNA Transcription Kit (Takara, Tokyo, Japan). qRT‐PCR was performed using a 7500 Real‐Time PCR system (Applied Biosystems, CA, USA) and SYBR Green reagent (Roche, Mannheim, Germany). Gene expression levels were normalized to that of β‐actin. The primer sequences were listed in Table S1 (Supporting Information).

### Western Blotting Analysis

The cells and EV fractions were lysed with RIPA buffer (Solarbio, Beijing, China). Protein concentrations were determined using a bicinchoninic acid (BCA) protein assay kit (Solarbio). Protein lysates were subjected to SDS‐PAGE and transferred to PVDF membranes (Millipore, Billerica, MA, USA). Membranes were blocked with 5% skim milk (Solarbio), incubated overnight at 4 °C with primary antibodies, followed by incubation with the corresponding horseradish peroxidase‐conjugated secondary antibodies. The intensity of each protein band was visualized using a Super ECL Plus kit (Affinibody Life Science, Switzerland). Normalization was performed by analyzing the same samples with antibodies against β‐actin. The antibodies used for western blotting were listed in Table S2 (Supporting Information).

### Flow Cytometry

After treatment with CM or EVs, macrophages were resuspended in PBS containing 2% FBS. The harvested cells were blocked with TruStain FcX (anti‐mouse CD16/32, Biolegend, San Diego, CA, USA) for 15 min on ice before stained with macrophage surface markers in the dark for 30 min at 4 °C. After staining, the cells were sorted using a flow cytometer (Beckman), and data were analyzed using FlowJo 10.8 software. The antibodies used for flow cytometry were listed in Table S2 (Supporting Information).

### Immunofluorescence Staining

The harvested cells were washed three times with PBS, fixed with 4% paraformaldehyde for 15 min. For permeabilization, the cells were incubated with a 0.1% Triton X‐100 (Sigma–Aldrich) solution for 10 min. The cells were then blocked with 5% goat serum (ZSGB‐BIO, Beijing, China) for 1 h. Primary antibodies (Table S2, Supporting Information) were added to the cells and incubated at 4 °C overnight, followed by incubation with the fluorescent dye‐conjugated secondary antibodies (Table S2, Supporting Information) for 1 h. Nuclei were counterstained with DAPI (Servicebio). Cells were viewed using a confocal laser scanning microscope (Olympus FV3000, Tokyo, Japan). Quantitative analysis of the fluorescence intensity values was performed by calculating the total intensity of the target proteins per unit area using ImageJ software.

### Isolation of EVs

When the cell confluency reached 85%, the medium was replaced with FBS‐free medium. Ctrl‐CM and Comp‐CM were collected and sequentially centrifuged at 300 g for 10 min, 2000 g for 10 min, and 10 000 g for 60 min to remove dead cells, cell debris, and organelles, respectively. After filtration through a 0.22‐µm filter (Millipore), the supernatant was ultracentrifuged at 100 000 g for 70 min at 4 °C (Beckman Coulter, Germany) to harvest EVs. The pellet was washed in PBS and ultracentrifuged again at 100 000 g for 70 min. After the supernatant was discarded, purified EVs derived from control cementoblasts (Ctrl‐EVs) and compression‐loaded cementoblasts (Comp‐EVs) were resuspended in PBS and stored at −80 °C for subsequent experiments. Before treatment with EVs, the culture medium of macrophages was replaced with FBS‐free DMEM.

### Identification of EVs

The protein concentration of EVs was determined using a BCA protein assay kit (Solarbio). Morphological identification was performed using TEM (JEM‐1400, JEOL, Japan). The size distribution and particle concentration of the EVs were determined using NTA (NS300, Malvern, UK). Furthermore, the expression of the EV‐specific markers Alix and CD63 was analyzed using western blotting.

### Cell Counting Kit‑8 (CCK‑8) Assay

Cementoblasts were subjected to different compression levels (0, 0.5, 1, 1.5, 2, 3 g cm^−^^2^) for 12 h. After removing the force‐loading device, cementoblasts were incubated with 450 µL fresh medium and 50 µL CCK‐8 reagent (Dojindo) at 37 °C for 90 min. To determine the proliferation of macrophages treated with Ctrl‐EVs or Comp‐EVs, cells were treated with Ctrl‐EVs or Comp‐EVs (10 or 30 µg mL^−1^) for 24 h, and evaluated at 0, 1, 2, 3 days using CCK‐8 reagent at 37 °C for 45 min. The absorbance was read at 450 nm using the ELX808 Plate Reader (BioTek, USA).

### Live/Dead Staining

Macrophages were treated with Ctrl‐EVs or Comp‐EVs (10, 30, 60, 100 µg mL^−1^) for 24 h. Then, the cells were stained with a calcein AM/PI double‐stain kit (Abbkine, Wuhan, China) according to the manufacturer's instructions. Live cells and dead cells were indicated by green and red fluorescence, respectively. All the samples were observed using a laser scanning microscope (Olympus). Cell viability was determined by calculating the ratio of the green fluorescence cells to the total number of cells.

### Fluorescent Labeling of EVs and Tracing EV Uptake by Macrophages

To trace EV uptake by macrophages, Ctrl‐EVs and Comp‐EVs were resuspended in DiI dye (Beyotime, Beijing, China) solution at 37 °C for 15 min in the dark to label red fluorescence. DiI‐labeled EVs were isolated via ultracentrifugation twice at 100 000 g for 70 min, and then co‐cultured with macrophages (RAW264.7) for 2 and 12 h. Afterward, the cells were washed with PBS to remove free EVs, and stained with F4/80 and DAPI to visualize the cytomembrane and nucleus, respectively. EV uptake was photographed using a confocal microscope (Olympus FV3000).

### Induction of Apoptosis and Fluorescent Labeling of Apoptotic Cells

OCCM‐30 and Jurkat cells were treated with 300 and 500 nM staurosporine, respectively, for 12 h to induce apoptosis. This method generated 90% Annexin V+ cells, as detected using an Annexin V‐FITC/PI Apoptosis Detection Kit (Dojindo). The apoptotic cells were resuspended at a concentration of 2 × 10^7^ cells mL^−1^ in DiI or DiO dye solution (Beyotime) for 15 min at 37 °C and then washed twice with DMEM for subsequent experiment.

### In Vitro Efferocytosis Assay

DiI+ or DiO+ apoptotic cementoblasts and lymphocytes were added to macrophages (RAW 264.7 or BMDMs) at a ratio of 5:1 for 45 min, followed by vigorous washing with PBS three times to remove unbound apoptotic cells. Next, the macrophages were stained with F4/80, and imaged with a confocal microscope (Olympus FV3000) or subjected to flow cytometry. For quantification, the ratios of DiI/DiO+ F4/80+ double‐positive macrophages were calculated, and the analysis was carried out using ImageJ software.

### Second Harmonic Generation (SHG) Imaging

To visualize collagen fibers, a two‐photon laser scanning confocal microscope (TCS‐SP8 DIVE, Leica, Germany) with SHG collection at 450 nm was used. FITC‐Phalloidin was used to label cementoblast. Without permeabilization with Triton X‐100, collagen I protein deposited in the ECM was visualized.

### Cell Transfection

Recombinant lentiviruses containing full‐length Rab35, and negative control vectors were purchased from Tsingke Biotech Co. Ltd. (Beijing, China) and transfection was performed according to manufacturer's instructions. OCCM‐30 cells were transduced with the lentivirus at a MOI of 100, using the empty vector as the control. Following a 4‐day selection with 2 µg mL^−1^ puromycin (Solarbio), the Rab35‐overexpressing cementoblasts and control cementoblasts were harvested. The efficiency of the stably transfected cell clones was confirmed using qRT‐PCR and western blotting.

### Alkaline Phosphatase Staining and Activity

Alkaline phosphatase (ALP) staining and activity assays were conducted after the induction of mineralization for 7 days. For ALP staining, cells were rinsed and fixed using 4% paraformaldehyde. The NBT/BCIP staining kit (Beyotime) was used according to the manufacturer's protocols, and the samples were then imaged using a scanner and a microscope. ALP activity was analyzed using an alkaline phosphatase assay kit (Beyotime). Briefly, cells were rinsed and lysed with lysis buffer. Cell lysates were incubated with p‐nitrophenyl phosphate (p‐NPP) solution at 37 °C for 30 min, and ALP activity was detected at 405 nm.

### Animals

Male C57BL/6J mice (22–25 g, 6–8 weeks old) and male Sprague–Dawley rats (200–220 g, 6–8 weeks old) were purchased from Vital River Laboratory Animal Technology Company (Beijing, China). All animal experimental protocols were conducted in compliance with the animal welfare ethical regulations and were approved by the Animal Use and Care Committee of Peking University (LA2023036). All animals were housed in a temperature‐ and humidity‐controlled specific pathogen‐free facility on a 12‐h light‐dark cycle with adequate water and food.

### Mouse Model of Root Resorption (RR)

C57BL/6J mice were randomly divided into four groups (n = 5 per group): 1) normal (untreated), 2) RR+PBS, 3) RR+Ctrl‐EVs, and 4) RR+Comp‐EVs. The mice were anesthetized via intraperitoneal injection of 0.5% pentobarbital sodium solution at a dose of 2.5 mL kg^−1^. The right maxillary first molar was ligated to the maxillary incisors using a nickel‐titanium coil spring (wire size, 0.2 mm; Smart Technology), providing a nearly constant force of ≈20 g for 3 weeks. For EV treatment, 10 µL of PBS, Ctrl‐EVs (1.5 µg µL^−1^) or Comp‐EVs (1.5 µg µL^−1^) were injected into the buccal gingiva of the first maxillary molar every 3 days using a 33‐gauge Hamilton syringe (Hamilton Company, USA). Mice were provided with soft food to ensure adequate energy intake. After 3 weeks, the mice were sacrificed for further experiments.

### The EV Release Profile of Hydrogel

The Gel/Ctrl‐EVs and Gel/Comp‐EVs composites, each containing 100 µg of EVs, were placed in a 96‐well plate. After gelation, the samples were soaked in 100 µL of double‐distilled water (ddH_2_O). Subsequently, the supernatant was collected, and the wells were refilled with 100 µL of ddH_2_O at 1, 3, 5, 7, 9, 11, and 13 days. The protein concentration in the collected supernatant was measured by BCA assay to draw a release curve.

### Preparation of EV‐hybrid Hydrogel

0.25% (w/v) LAP and gelatin methacryloyl (GelMA; EFL‐GM‐90, Yongqinquan Intelligent Equipment Co., Ltd.) were mixed for 30 min at 70 °C until completely dissolved to obtain a 20% (w/v) GelMA‐hybrid hydrogel. Next, 25 µL of Ctrl‐EVs or Comp‐EVs (4 µg µL^−1^) were resuspended in 75 µL of GelMA‐hybrid hydrogel at 37 °C to produce the Gel/EV mixed solutions, which were then applied to the calvarial defect areas.

### Rat Calvarial Defect Model

Sprague–Dawley rats were randomly divided into four groups (n = 5 per group): 1) blank, 2) Gel, 3) Gel/Ctrl‐EVs, and 4) Gel/Comp‐EVs. The rats were anesthetized via intraperitoneal injection of 1% pentobarbital sodium solution at a dose of 2.5 mL kg^−1^. After exposing the calvarium, two critically sized, full‐thickness calvarial defects (5 mm in diameter) were created symmetrically using a trephine bur under copious sterile saline irrigation. Different Gel/EV mixed solutions were then gently implanted into the calvarial defect according to each group's assignment and subsequently exposed to ultraviolet (UV) light for 30 s for photopolymerization. The soft tissues were then closed, and the skin was sutured. The animals were euthanized 12 weeks after surgery for tissue evaluation.

### Microcomputed Tomography (micro‐CT) Analysis

All fixed samples were scanned using a high‐resolution micro‐CT system (SkyScan 1276; Bruker, Belgium). Mice maxilla samples were scanned at a voltage of 85 kV, a current of 65 µA, and a voxel resolution of 3.5 µm. Rat calvarium samples were scanned at a voltage of 70 kV, a current of 200 µA, and a voxel resolution of 13 µm. Micro‐CT scanning data were transferred to SkyScan CT Analyzer (version 1.17.7.2) and Mimics (version 21.0) software (Materialize, Leuven, Belgium) for 3D image reconstruction. The calculation of root resorption volume was performed using Mimics software. The resorption lacunae on the compression side of the distobuccal roots were manually smoothed according to the control side to approximate the natural contour of the roots. The volume of root resorption was calculated by subtracting the post‐resorption root volume from the intact root volume. The ratio of new bone area to total defect area was calculated using ImageJ software. The percentage of new bone volume/total tissue volume (BV/TV) and bone mineral density (BMD) were analyzed using CTAn software.

### Histological and Immunohistochemical Staining

All samples were demineralized in 10% ethylenediaminetetraacetic acid (EDTA) for at least three weeks, embedded in paraffin, and cut into 5‐µm‐thick sections. At least three sections from each sample were collected and stained with hematoxylin and eosin (Solarbio) and Masson's trichrome (Servicebio) to evaluate histological alterations. Tartrate‐resistant acid phosphatase TRAP staining (Sigma–Aldrich) was used to evaluate osteoclast activity in compression‐loaded cementum and alveolar bone. Each section was observed under a light microscope (Carl Zeiss, Jena, Germany). The number of TRAP‐positive cells was then counted.

For immunohistochemical staining, sections were deparaffinized, and antigen retrieval was performed by boiling the samples in a citrate buffer solution (pH 6.0) at 95 °C for 8 min. After incubation with the primary antibodies (Table S2, Supporting Information), the samples were incubated with the corresponding horseradish peroxidase‐conjugated secondary antibodies (Servicebio). Immunohistochemical staining was observed under a microscope (Carl Zeiss), and quantitative analysis was performed using ImagePro Plus 6.0 software.

### Tissue Immunofluorescence Staining

Sections were deparaffinized, and subjected to antigen retrieval. They were blocked with 5% goat serum (Solarbio) for 1 h, and incubated with the primary antibodies (Table S2, Supporting Information) overnight at 4 °C. Following incubation, the sections were washed with PBS and subsequently incubated with corresponding fluorescence‐conjugated secondary antibodies (Table S2, Supporting Information) for 1 h. The nuclei were counterstained with DAPI (Servicebio). Fluorescence images were captured using a fluorescence microscope (Olympus FV3000), and the fluorescence intensity was quantified using ImageJ software.

### Terminal Deoxynucleotidyl Transferase‐mediated dUTP‐biotin Nick‐end Labeling (TUNEL) Assay

To assess apoptosis on the compression side of roots, TUNEL staining was performed using a TUNEL apoptosis assay kit (Servicebio) according to the manufacturer's instructions. Sections were observed under a confocal laser microscope (Olympus FV3000).

### Statistical Analysis

All data are presented as the mean ± standard deviation (SD). GraphPad Prism 9.0 (GraphPad, USA) was used for statistical analysis. The two‐tailed Student's *t*‐test was used to determine significance between the two groups. Comparisons among multiple groups were performed using one‐way analysis of variance (ANOVA), followed by Dunnett's post hoc test. All experiments were repeated at least three times. The threshold for statistical significance was established at *P* < 0.05.

## Conflict of Interest

The authors declare no conflict of interest.

## Author Contributions

W.L. and Y.H. conceived and supervised the project. W.L., Y.H., and Y.Y. designed experiments. Y.Y. performed most of the experiments and analyzed the data. H.L. and K.G. performed the animal experiments. Q.Y., Y.Z., and J.W. analyzed the data. Y.Y., Y.H., and W.L. wrote the manuscript.

## Supporting information

Supporting Information

## Data Availability

The data that support the findings of this study are available from the corresponding author upon reasonable request.
