# Correction to “Extracellular Vesicles from Compression‐Loaded Cementoblasts Promote the Tissue Repair Function of Macrophages”

**DOI:** 10.1002/advs.202501438

**Published:** 2025-03-05

Adv Sci (Weinh) **2024**, *11*(36), e2402529.

https://doi.org/10.1002/advs.202402529

In the original version of Figure 1I, the “iNOS” image of the Blank group was mistakenly represented as the image of the Ctrl‐EVs group in Figure 2E. The corrected figure is shown below. As the quantification analysis was conducted using the correct images, the results remain unchanged.

Corrected Figure 1I


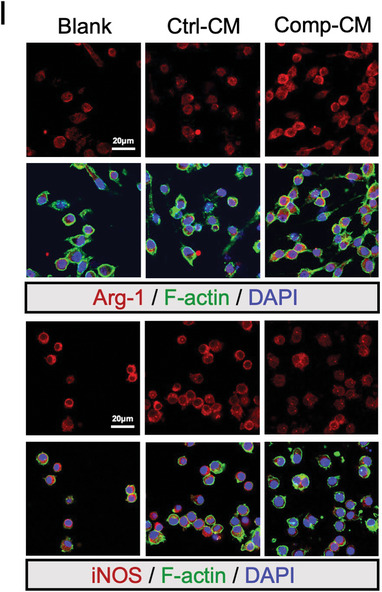


In Figure 4G, the OCN images of the “RR+Ctrl‐EVs” and “RR+Comp‐EVs” groups were improperly used. The corrected figure is presented below with updated quantification.

Corrected Figure 4G


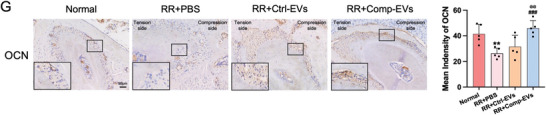


In Figure S9 (Supporting Information), the image of CD163 in the “RR+PBS” group was incorrectly assembled. The corrected figure is presented below.

Corrected Figure S9


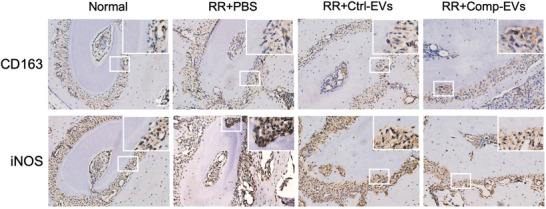


These corrections do not impact the overall findings or conclusions of the paper. We sincerely apologize for these errors.

