# Supplementary material for: Extracellular Vesicles from Compression‐Loaded Cementoblasts Promote the Tissue Repair Function of Macrophages
Source: Adv Sci (Weinh). 2024 Aug 5;11(36):2402529. doi: 10.1002/advs.202402529 (PMC11423227; doi:10.1002/advs.202402529)
Supplement: Supplementary file 1 — Supporting Information [file ADVS-11-2402529-s001.pdf]

ADVANCED SCIENCE
Open Access

Supporting Information

for *Adv. Sci.*, DOI 10.1002/advs.202402529

Extracellular Vesicles from Compression-Loaded Cementoblasts Promote the Tissue Repair Function of Macrophages

Yuhui Yang, Hao Liu, Kunyao Guo, Qianyao Yu, Yi Zhao, Jiayi Wang, Yiping Huang and Weiran Li**

Extracellular Vesicles from Compression-Loaded Cementoblasts Promote the Tissue Repair Function of Macrophages

Yuhui Yang, Hao Liu, Kunyao Guo, Qianyao Yu, Yi Zhao, Jiayi Wang, Yiping Huang, Weiran Li**

Supplementary Figures

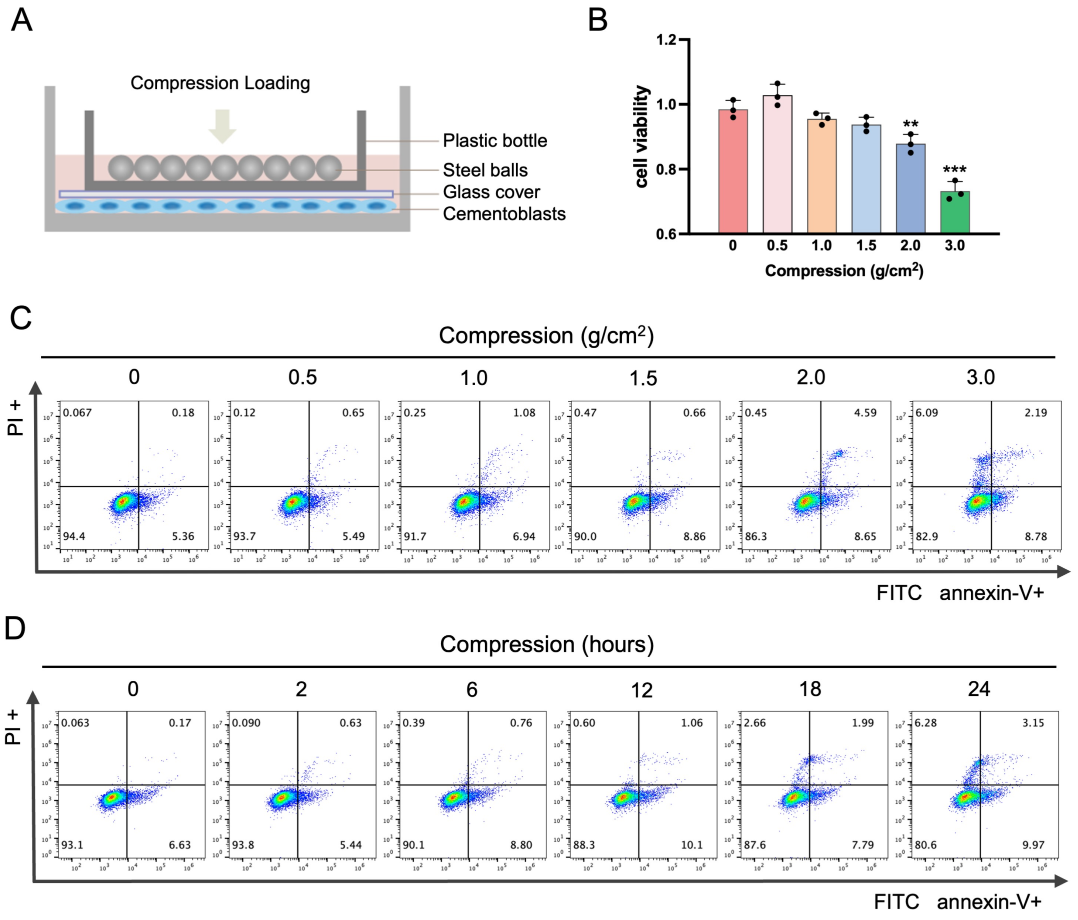

Figure S1. The viability and apoptosis of cementoblasts subjected to compression at different magnitudes and durations. (A) Schematic illustration of the compression-loading device. (B) Cell viability of cementoblasts under different compression levels (0, 0.5, 1, 1.5, 2, 3 g cm^{-2}) for 12 h examined by CCK-8 assay (n = 3). (C) The ratio of apoptotic or necrotic cementoblasts under different compression levels (0, 0.5, 1, 1.5, 2, 3 g cm^{-2}) for 12 h, measured by Annexin-V and PI staining (n = 3). (D) The ratio of apoptotic/necrotic cementoblasts under compression at 1.5 g cm^{-2} for different durations (0, 2, 6, 12, 18, and 24 h) (n = 3). The data are presented as the mean ± SD. *P < 0.05, **P < 0.01 and ***p < 0.001.

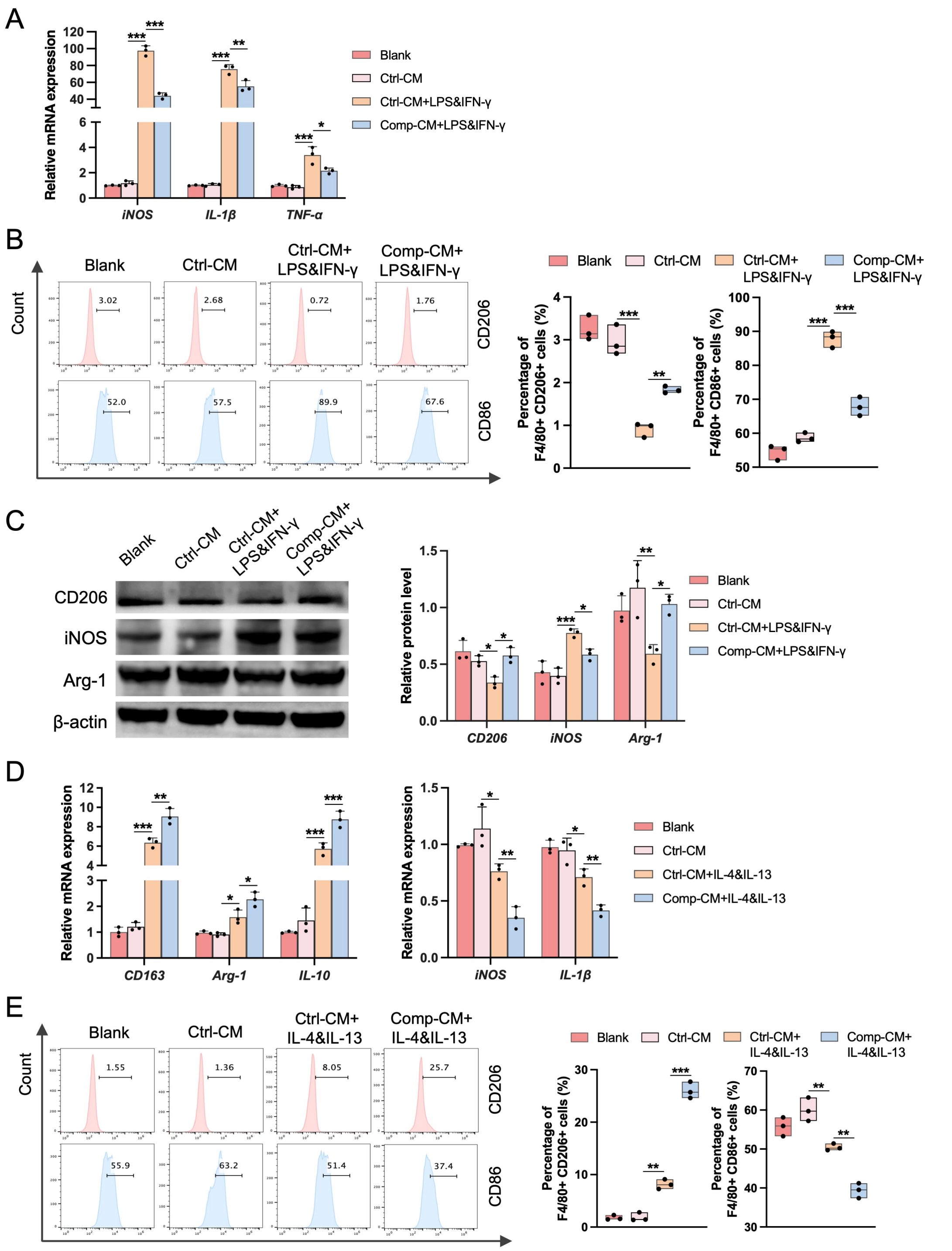

Figure S2. Conditioned media from compression-loaded cementoblasts (Comp-CM) promote the polarization of LPS & IFN-γ-induced or IL-4 & IL-13-induced macrophages toward the M2 type. (A) Relative mRNA expression levels of *iNOS, IL-1β*, and *TNF-α* in macrophages (RAW 264.7) treated with PBS or LPS & IFN-γ for 24 h, and then cultured with blank media, Ctrl-CM, or Comp-CM for 24 h (n = 3). (B) Flow cytometry analysis of M2 and M1 populations in F4/80+ macrophages.

Quantification of the percentage of CD206+ and CD86+ macrophages (n = 3). (C) The protein levels of CD206, Arg-1, and iNOS, and quantitative analysis in terms of the band intensity (n = 3). (D) Relative mRNA expression levels of M2 and M1 markers in macrophages treated with PBS or IL-4 & IL-13 for 24 h, and then cultured with blank media, Ctrl-CM, or Comp-CM for 24 h (n = 3). (E) Flow cytometry analysis of M2 and M1 populations in F4/80+ macrophages. Quantification of the percentage of CD206+ and CD86+ macrophages (n = 3). The data are presented as the mean ± SD. *P < 0.05, **P < 0.01 and ***p < 0.001.

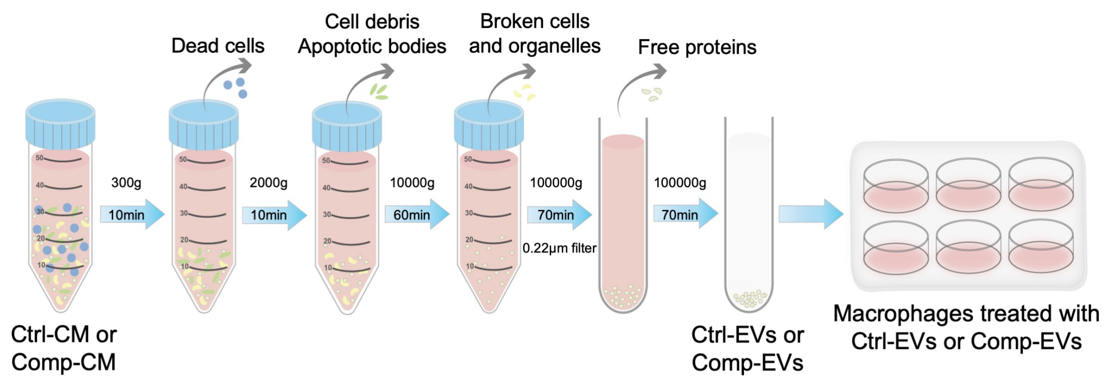

Figure S3. Schematic diagram of the isolation of the extracellular vesicles from compression-loaded cementoblasts (Comp-EVs) and control cementoblasts (Ctrl-EVs).

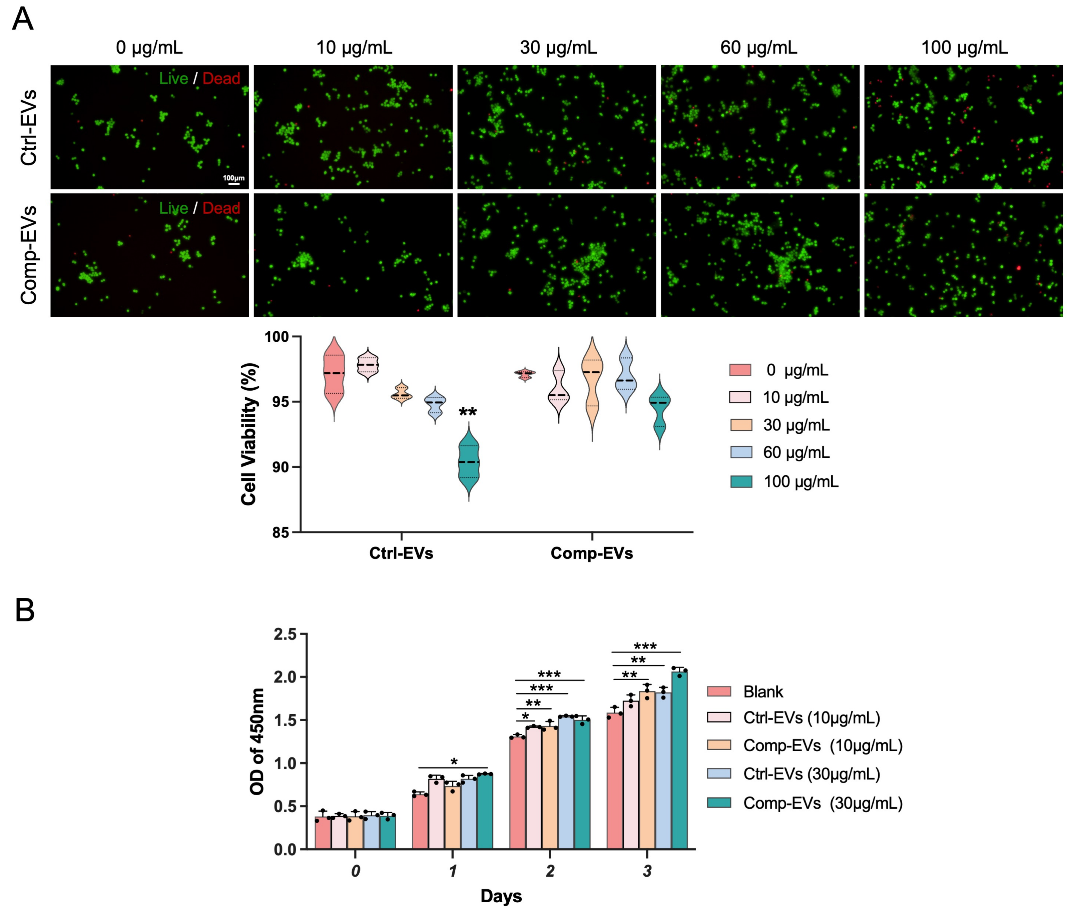

Figure S4. The viability of cementoblasts treated with Comp-EVs and Ctrl-EVs. (A) Representative live/dead staining images of macrophages treated with Ctrl-EVs or Comp-EVs at different concentrations (0, 10, 30, 60, 100 μg mL^{-1}) for 24 h. Quantification of the ratio of live cells to total cells (n = 3). (B) CCK-8 assay of macrophages after treatment with Ctrl-EVs or Comp-EVs (n = 3). The data are presented as the mean ± SD. *P < 0.05, **P < 0.01 and ***p < 0.001.

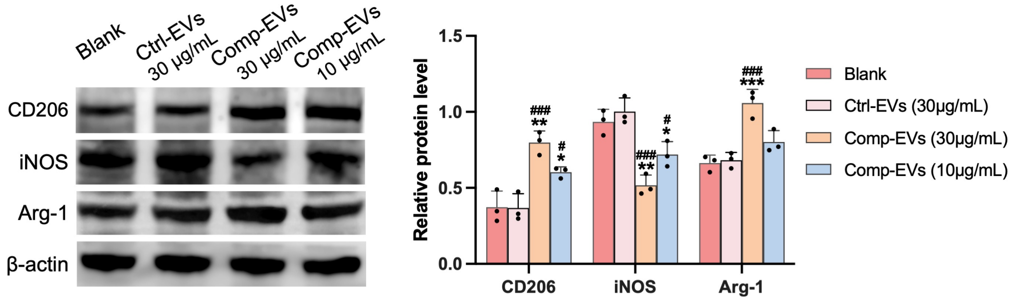

Figure S5. The protein levels of CD206, Arg-1 and iNOS in macrophages treated with PBS, Ctrl-EVs (30 μg mL^{-1}), Comp-EVs (30 μg mL^{-1}), and Comp-EVs (10 μg mL^{-1}) for 24 h. Semiquantitative analysis in terms of the band intensity (n = 3). The data are presented as the mean ± SD. *$P < 0.05$, **$P < 0.01$ and ***$p < 0.001$ versus blank group; $^{\#}p < 0.05$, $^{\#\#}p < 0.01$, and $^{\#\#\#}p < 0.001$ versus Ctrl-EVs group.

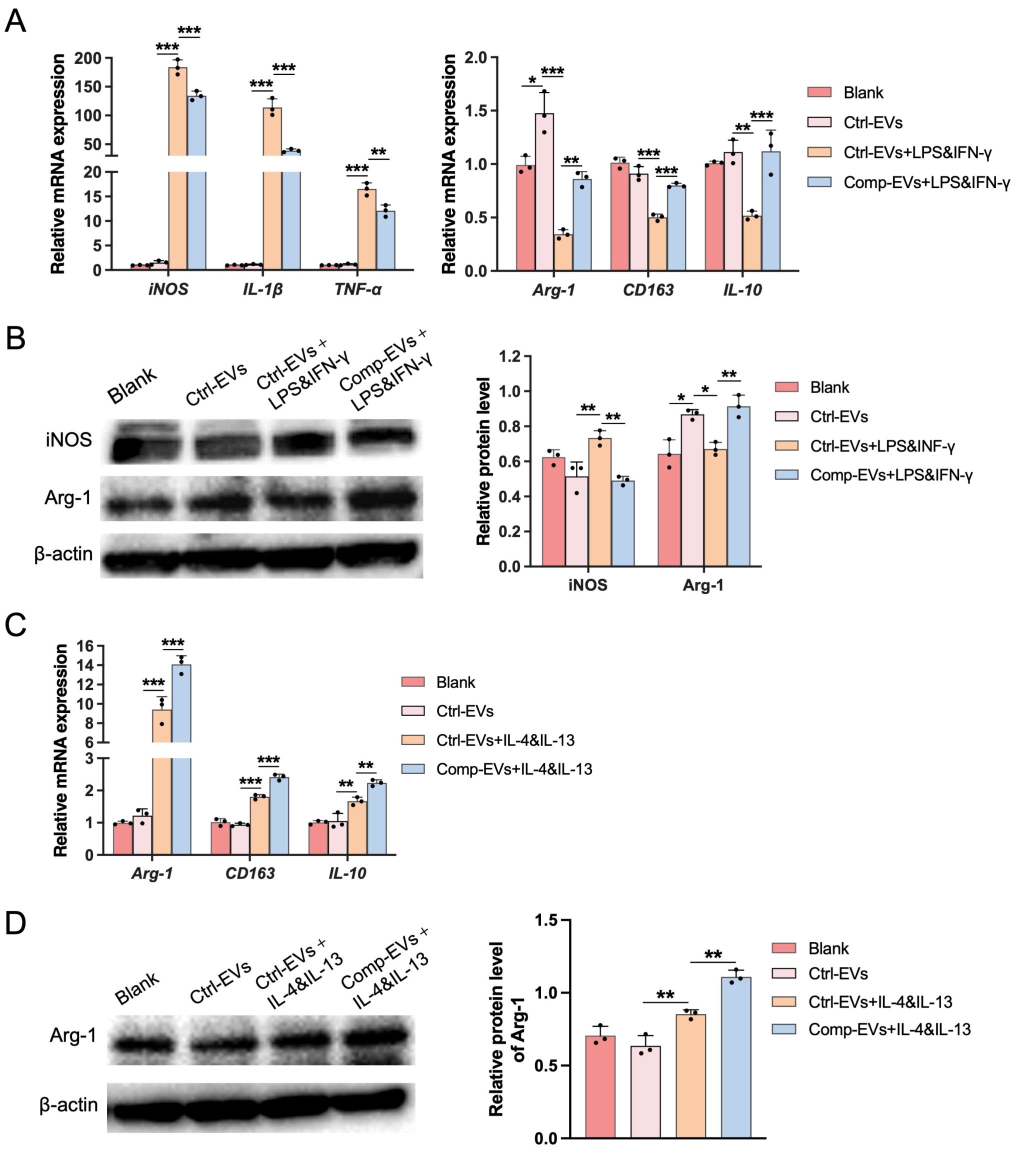

Figure S6. Comp-EVs promote the polarization of LPS & IFN-γ-induced or IL-4 & IL-13-induced macrophages toward the M2 type. (A) Relative mRNA expression levels of M1 and M2 markers in macrophages treated with PBS or LPS & IFN-γ for 24 h, and then treated with PBS, Ctrl-EVs (30 μg mL^{-1}), or Comp-EVs (30 μg mL^{-1}) for 24 h (n

= 3). (B) The protein levels of Arg-1 and iNOS. Semiquantitative analysis of the band intensity (n = 3). (C) Relative mRNA expression levels of *Arg-1*, *CD163*, and *IL-10* in macrophages treated with PBS or IL-4 & IL-13 for 24 h, and then treated with PBS, Ctrl-EVs (30 μg mL^{-1}), or Comp-EVs (30 μg mL^{-1}) for 24 h (n = 3). (D) The protein level of Arg-1 and semiquantitative analysis of the band intensity (n = 3). The data are presented as the mean ± SD. *P < 0.05, **P < 0.01 and ***p < 0.001.

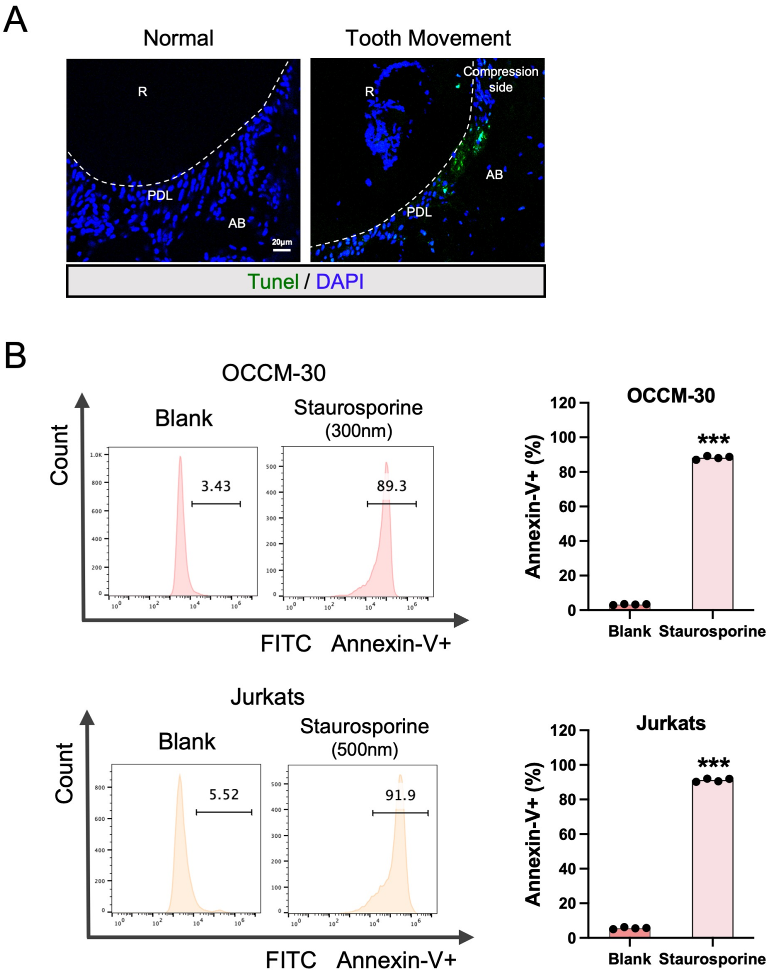

Figure S7. (A) Representative images of TUNEL staining on the compression side of roots. R, root; PDL, periodontal ligament; AB, alveolar bone. Scale bar: 20μm. (B) The ratio of Annexin-V+ apoptotic cementoblasts and lymphocytes induced by staurosporine (STS) for 12 h (n = 4). The data are presented as the mean ± SD. ***p < 0.001.

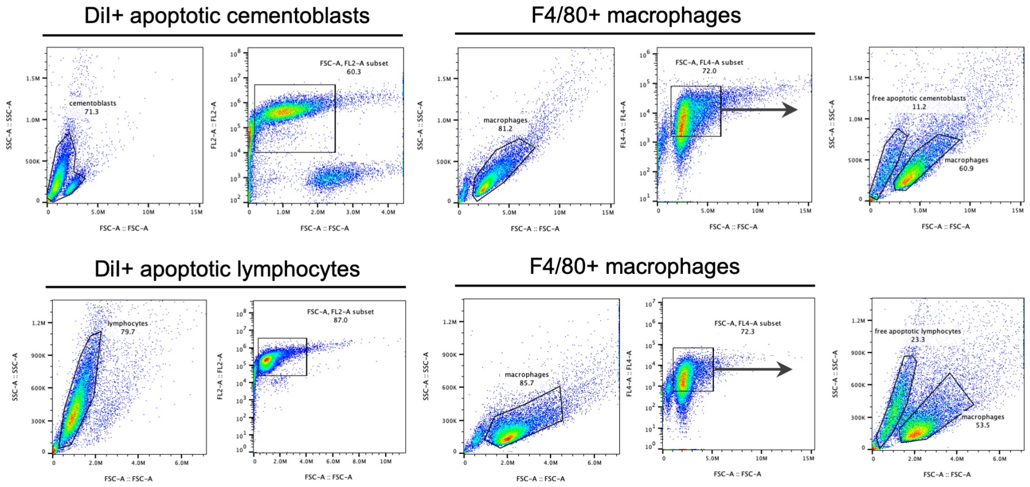

Figure S8. Flow cytometry gating scheme used to identify DiI+ cementoblasts or lymphocytes, and F4/80+ macrophage population.

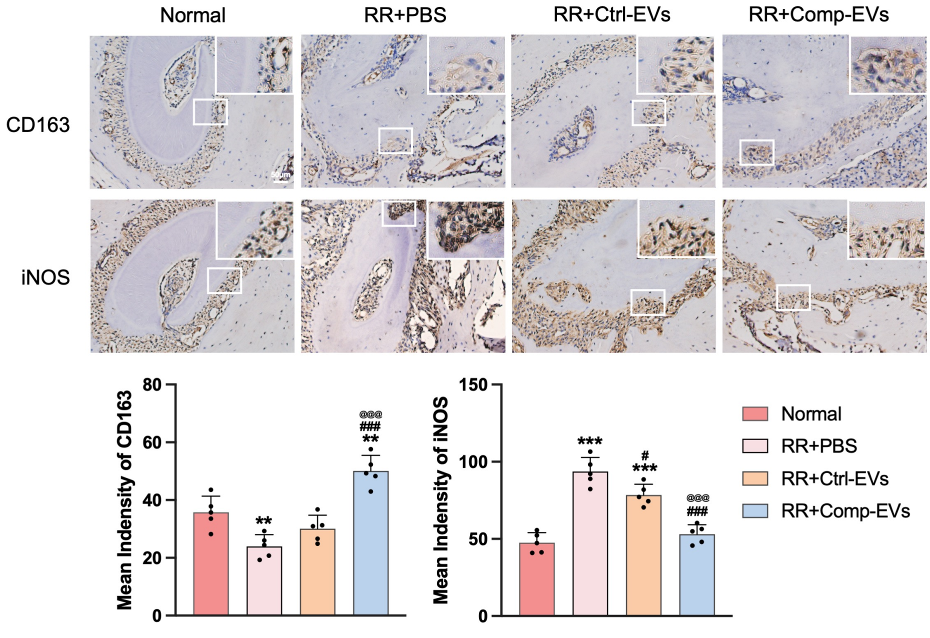

Figure S9. Representative immunohistochemical staining images of CD163 and iNOS on the compression side of roots and quantitative analysis (n = 5). Scale bars: 50 μm. The data are presented as the mean ± SD. $^{*}p < 0.05$, $^{**}p < 0.01$, and $^{***}p < 0.001$ versus normal group; $^{\#}p < 0.05$, $^{\#\#}p < 0.01$, and $^{\#\#\#}p < 0.001$ versus RR+PBS group; $^{@}p < 0.05$, $^{@@}p < 0.01$, and $^{@@@}p < 0.001$ versus RR+Ctrl-EVs group.

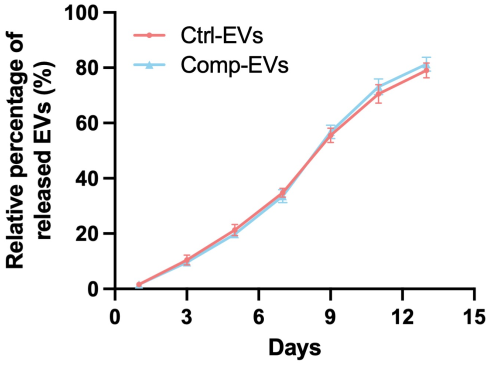

Figure S10. Cumulative relative release quantities of Ctrl-EVs and Comp-EVs at days 1, 3, 5, 7, 9, 11, and 13 (n = 3). The data are presented as the mean ± SD.

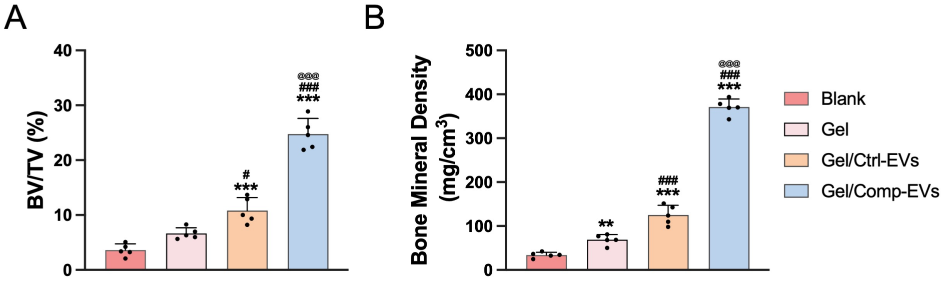

Figure S11. Histomorphometric analysis of BV/TV and BMD parameters of calvarial defects in the blank, Gel, Gel/Ctrl-EVs, and Gel/Comp-EVs groups (n = 5). The data are presented as the mean ± SD. *p < 0.05, **p < 0.01, and ***p < 0.001 versus blank group; $^{\#}$p < 0.05, $^{\#\#}$p < 0.01, and $^{\#\#\#}$p < 0.001 versus Gel group; $^{@}$p < 0.05, $^{@@}$p < 0.01, and $^{@@@}$p < 0.001 versus Gel/Ctrl-EVs group.

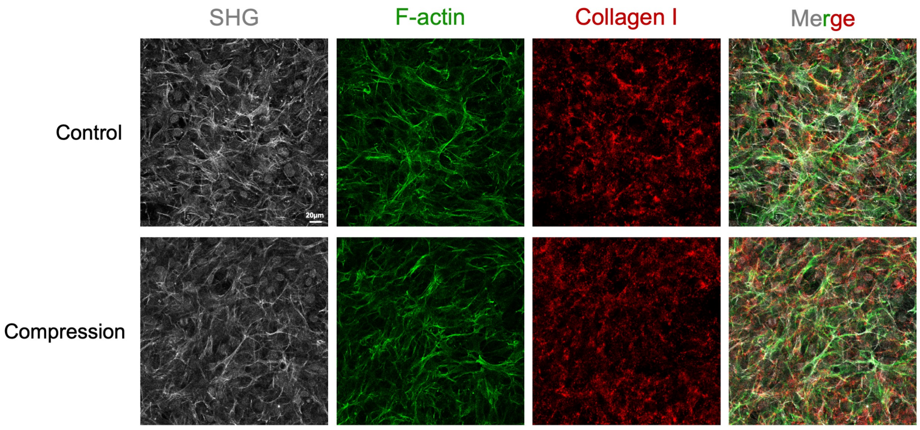

Figure S12. Cementoblasts were subjected to compression (1.5 g cm^{-2}, 12 h) and incubated in mineralized medium for 4 days. Imaging of second-harmonic generation (SHG, white), F-actin (green), and Collagen I (red) signals in compression-loaded cementoblasts and control cementoblasts. Scale bars: 20 μm.

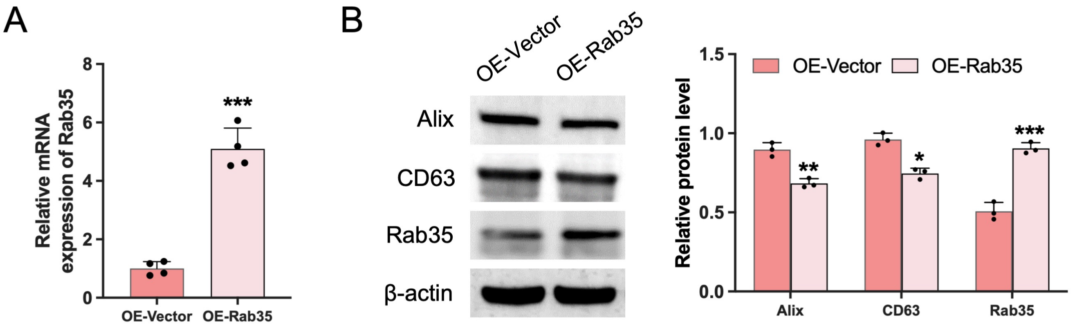

Figure S13. Construction of Rab35-overexpression cementoblasts. (A) The relative mRNA expression level of *Rab35* in Rab35-overexpressing cementoblasts and control cementoblasts (n = 4). (B) The protein levels of Alix, CD63, and Rab35, along with semiquantitative analysis (n = 3). The data are presented as the mean ± SD. *P < 0.05, **P < 0.01 and ***p < 0.001.

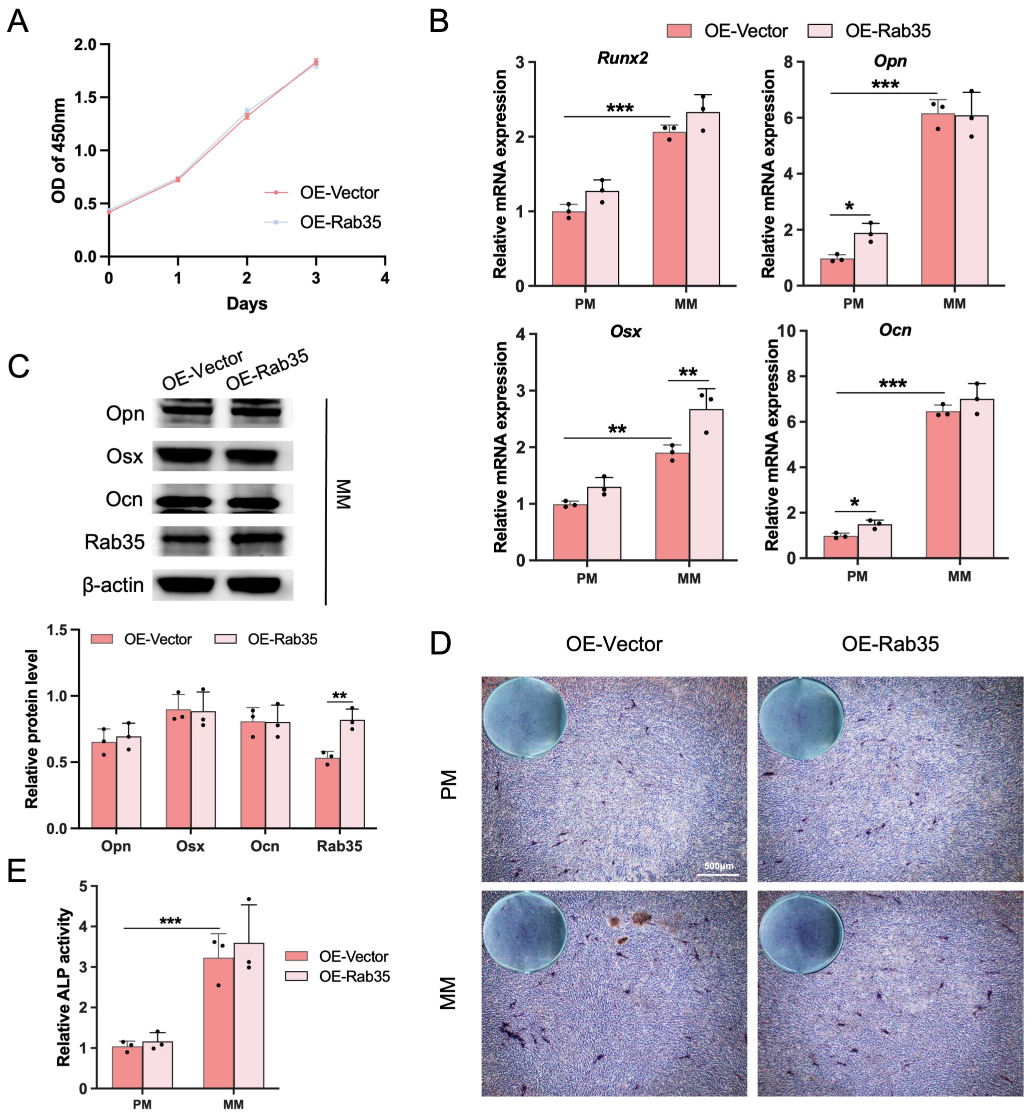

Figure S14. Mineralization capability of Rab35-overexpressing cementoblasts and control cementoblasts. (A) CCK-8 assay of Rab35-overexpression cementoblasts and control cementoblasts (n = 3). (B) Relative mRNA expression levels of *Runx2*, *Opn*, *Osx*, and *Ocn* in Rab35-overexpression cementoblasts and control cementoblasts after 7 days of proliferation or mineralization induction (n = 3). (C) The protein levels of Opn, Osx, Ocn, and Rab35, with semiquantitative analysis of band intensity (n = 3). (D, E) ALP staining and activity in Rab35-overexpression cementoblasts and control cementoblasts (n = 3). Scale bar: 500 μm. The data are presented as the mean ± SD. *P < 0.05, **P < 0.01 and ***p < 0.001.

Supplementary Tables

Table S1. Sequences of primers used in qRT-PCR

Gene	Forward primer sequence (5′–3′)	Reverse primer sequence (5′–3′)
Arg-1	CTCCAAGCCAAAGTCCTTAGAG	AGGAGCTGTCATTAGGGACATC
CD163	GGCTAGACGAAGTCATCTGCAC	CTTCGTTGGTCAGCCTCAGAGA
IL-10	AAGGGTTACTTGGGTTGCCA	CCTGGGGCATCACTTCTACC
PPARγ	TCGCTGATGCACTGCCTATG	GAGAGGTCCACAGAGCTGATT
iNOS	GGAGTGACGGCAAACATGACT	TCGATGCACAACTGGGTGAAC
TNF-α	CCTGTAGCCCACGTCGTAG	GGGAGTAGACAAGGTACAACCC
IL-1β	GAAATGCCACCTTTTGACAGTG	TGGATGCTCTCATCAGGACAG
Rab35	ACGATTCGCAGACAACACCT	GCAGCAGCGTTTCTTTCGTT
Rab27a	TCGGATGGAGATTACGATTACCT	TTTTCCCTGAAATCAATGCCCA
Rab27b	CGTCAGGAAAAGCGTTTAAGGT	AGAAGCTCTGTTGACTGGTGA
Osx	ATGGCGTCCTCTCTGCTTG	TGAAAGGTCAGCGTATGGCTT
Ocn	ACCCTGGCTGCGCTCTGTCTCT	GATGCGTTTGTAGGCGGTCTTCA
Runx2	ATGCTTCATTCGCCTCACAAA	GCACTCACTGACTCGGTTGG
Opn	TTTACAGCCTGCACCC	CTAGCAGTGACGGTCT
β-actin	AGGGAAATCGTGCGTGACAT	TCCAGGGAGGAAGAGGATGC

Table S2. Antibodies used for western blotting, flow cytometry, and immunofluorescence staining

Antibody	Source	Identifier
Mouse anti-Arg-1	Santa Cruz	Cat# sc-271430 (1:50 dilution)
Rabbit anti-Arg-1	Proteintech	Cat# 16001-1-AP (1:1000 dilution)
Mouse anti-iNOS	Santa Cruz	Cat# sc-7271 (1:50 dilution)
Rabbit anti-iNOS	Proteintech	Cat# 22226-1-AP (1:1000 or 1:200 dilution)
Rabbit anti-Alix	Proteintech	Cat# 12422-1-AP (1:1000 or 1:200 dilution)
Mouse anti-CD63	Santa Cruz	Cat# sc-5275 (1:50 dilution)
Rabbit anti-CD163	Servicebio	Cat# GB113751-100 (1:200 dilution)
Rabbit anti-CD206	Proteintech	Cat# 18704-1-AP (1:800 dilution)
Rabbit anti-iNOS	Servicebio	Cat# GB11119-100 (1:200 dilution)
Rabbit anti-OCN	Servicebio	Cat# GB11233-100 (1:200 dilution)

Mouse anti-CD31	Servicebio	Cat# GB12063-100 (1:200 dilution)
Rabbit anti-F4/80	Proteintech	Cat# 28463-1-AP (1:200 dilution)
Rabbit anti-β-actin	Proteintech	Cat# 66009-1-Ig (1:2000 dilution)
Mouse anti-Cap	Santa Cruz	Cat# sc-53947 (1:50 dilution)
Rabbit anti-Rab35	ABclonal	Cat# A8030 (1:1000 or 1:200 dilution)
Rabbit anti-Osx	Abcam	Cat# ab209484 (1:1000 dilution)
Rabbit anti-Opn	Proteintech	Cat# 22952-1-AP (1:1000 dilution)
Rabbit anti-Ocn	ABclonal	Cat# A6205 (1:1000 dilution)
Rabbit anti-Collagen I	Proteintech	Cat# 14695-1-AP (1:200 dilution)
APC anti-mouse CD86	Biolegend	Cat# 105113 (1:100 dilution)
PE anti-mouse CD163	Biolegend	Cat# 155307 (1:100 dilution)
APC anti-mouse F4/80	Biolegend	Cat# 123116 (1:100 dilution)
FITC anti-mouse F4/80	Biolegend	Cat# 123107 (1:100 dilution)
PE anti-mouse CD206	Biolegend	Cat# 141705 (1:100 dilution)
APC mouse IgG1κ	Biolegend	Cat# 400119 (1:100 dilution)
PE anti-mouse IgG1	Biolegend	Cat# 406607 (1:100 dilution)
FITC Phalloidin	ABclonal	Cat# RM02836 (1:200 dilution)
Goat anti-mouse (HRP)	ZSGB-BIO	Cat# ZB-2305 (1:8000 dilution)
Goat anti-rabbit (HRP)	ZSGB-BIO	Cat# ZB-2301 (1:8000 dilution)
Goat anti-rabbit (AF488)	ZSGB-BIO	Cat# ZF-0511 (1:200 dilution)
Goat anti-rabbit (CL594)	Proteintech	Cat# SA00013-4 (1:200 dilution)
Goat anti-mouse (AF555)	Huabio	Cat# HA1118 (1:200 dilution)
